# Expenditure projections for community home-based care services for older adults with functional decline in China

**DOI:** 10.1186/s12939-023-01954-y

**Published:** 2023-07-29

**Authors:** Ying Han, Chuanhai Xu, Liangwen Zhang, Yafei Wu, Ya Fang

**Affiliations:** 1 State Key Laboratory of Molecular Vaccinology and Molecular Diagnostics, School of Public Health, Xiamen University, Xiang’an South Road, Xiamen, Fujian 361102 PR China; 2 School of Economics, Xiamen University, 422 Siming South Road, Xiamen Fujian, 361005 PR China

**Keywords:** Community home-based care, Markov model, Man-hour costing, Project cost method, Functional decline

## Abstract

**Introduction:**

Difficulty in identifying the functional status of older adults creates an imbalance between the supply and demand for community home-based care. Using a multi-level functional classification system to guide care cost measurement may optimize care resources and meet diverse eldercare demands.

**Methods:**

The Markov model was used to project the older population size in different functional decline (FD) statuses. The project cost and the man-hour costing method were combined to forecast the cost of community home-based care for older adults with FD.

**Results:**

The projected cost of eldercare increased from 1668.623 billion yuan in 2020 to 2836.754 billion yuan in 2035. By 2035, the total cost for community-based home care for those in pathological development of FD statuses such as “viability disorder,” “acute disease,” “somatic functional disorder,” and “sub-disorder” was projected to be 1094.591 billion, 433.855 billion, 1256.236 billion, and 52.072 billion yuan, respectively, which is 1.24, 1.58, 1.78, and 0.49 times higher than the results by the man-hour costing method. Family caregiving costs are about three times those of professional caregivers.

**Conclusion:**

The escalating cost of providing graded care for older adults, particularly by family caregivers, presenting a significant evidence for the need to optimize resource allocation and develop a robust human resources plan for community home-based care.

**Supplementary Information:**

The online version contains supplementary material available at 10.1186/s12939-023-01954-y.

## Main points

The projections of expenditure by both the project accounting method and the man-hour costing method. The highest costs for urinary catheterization, stoma care, nasal feeding, and pressure ulcer prevention care. The cost of community home-based care for older people with FD will further increase. A large gap will still exist in the cost needs of community home-based older adults for home care and professional care.

## Introduction

Population aging is a significant global social issue, with more than 142 million older people (representing 14% of the global population aged 60 years and above) estimated to be unable to meet their basic daily needs [1]. In China, the proportion of individuals aged over 60 is expected to increase from 18.7% to 2020 to 40.0% in 2070, surpassing most European Union countries [2, 3]. The prevalence of diseases among the older population has led to a surge in demand for eldercare services. Older individuals often experience a decline in physical function and loss of independent living ability, leading to a rise in health problems. Around 18.6% of disabled older people, and about 90.6% of older adults with chronic diseases both require care from family and society [4]. These demographic changes will significantly impact future healthcare utilization and expenditures [5]. Moreover, it is predicted that the consumer demand for China’s eldercare industry market will reach 8 trillion yuan and 22 trillion RMB respectively, by 2020 and 2030, and the pension gap will reach 8 trillion RMB to 10 trillion RMB [6]. However, various types of pension funds in China only accumulate to 12 trillion RMB, which cannot meet the market demand. The proportion of pension savings to income is only 27%, and personal savings for pension funds have not been effectively converted into pension wealth reserves [7]. The national pension wealth reserve is still insufficient, highlighting a discrepancy between the supply and demand for eldercare and an accelerated increase in care costs.

To address the challenges of population aging, the Chinese government has proposed the development of community home-based care services. These services aim to meet the growing needs of the older population for long-term care and reduce health expenditures by lowering hospitalization rates and the number of hospital days, thus alleviating the burden of healthcare [8]. This approach aligns with the World Health Organization’s (WHO) recommendation to foster the abilities of older people within their communities. Community home-based care services refer to continuous and comprehensive medical care, social support, and daily life care services provided to people with disabilities or chronic diseases in a home environment by healthcare professionals and family caregivers [9]. Despite the growing popularity of community home-based care services for older adults, significant challenges remain in improving the quality and scope of care, controlling costs, coordinating healthcare professionals and family caregivers, and increasing the availability and accessibility of services, particularly in rural and remote areas [10]. Informal or unpaid caregiving by family members is often overlooked in contrast to formal caregiving provided by professionals for pay [11]. Currently, community home-based care for older adults in China is in the initial exploration stage, and there is no uniform cost calculation standard or regulation. There are significant differences in policy development among pilot cities, and both the government and society face financial constraints, as well as inadequate eldercare reserves. Thus, accurately calculating the cost of eldercare services and analyzing their changing trend has become a key challenge in China’s social care system. This will help to identify the gaps in eldercare funding, develop reasonable fundraising plans, and allocate resources effectively.

Establishing an eldercare cost standard requires the classification of functional grades as a prerequisite. The selection of functional classification index and content is crucial for accurately predicting the cost of community home-based care [12, 13]. However, there is currently a lack of comprehensive criteria for assessing the multi-level health statuses of older persons [14]. Some scholars have used different measures to classify care demand levels, including utilization of resources of community home-based care services, physical functional statuses of older adults with dementia, and activities of daily living (ADLs) assessment [15–18]. However, there is a lack of consideration for the continuum function of older adults, ranging from less independent to disabled. Functional capacity (FC) has been defined as an individual’s ability to perform survival-related activities autonomously [19, 20], and is a valid geriatric health assessment [21]. It is a reflection of various diseases, physical and mental conditions, and is a major factor causing the diversified needs of older adults [1, 22]. Thus FC can be used to assess an older person’s combined physical, emotional, and social interaction abilities, with the pathological development of functional decline (FD), followed by disability in activities of daily living (ADLs) [23].

The Markov model is the primary research method used for demand forecasting of long-term care [17]. This model constructs a set of discrete random variables with Markovian properties that track the evolution of the time continuum by considering only the present state and not the past state [24]. Sun applied the Markov model to predict older population sizes and human resources for eldercare and further verified it with the latest survey data of the China Health and Retirement Longitudinal Study (CHARLS) [25]. The Markov model has simple data requirements, is easy to handle statistically, and can predict the size of the older population and human resources needed for nursing care in China with high accuracy.

Therefore, this study aimed to refine the methodologies for predicting the demand and content of care services for community home-based care needs for older adults, as well as their population size and the cost of service programs at different levels. To achieve this goal, we attempted to address the following questions: (1) How does the multi-dimensional FC statuses of older adults taking community home-based care change? (2) What future care services programs will be needed for older adults with different states of FD? (3) What costs will be incurred for eldercare? To address the above questions, the study first classified the multi-dimensional FC of the older population according to personal, resource, and social indicators based on Maslow’s hierarchy of needs and Amartya Sen’s “feasible ability” theory. Second, the Markov model was used to measure the probability matrix of the transition of older adults with functional decline statuses within five years, based on CHARLS data from 2011, 2013, and 2015. Then, the number of older individuals with different statuses of functional decline in China in 2035 was projected using the Markov model and data on China’s older population aged 60 years or over from the United Nations Population Division. Lastly, the cost of community home-based care services from 2015 to 2035 was projected using both project costs and the man-hour costing method. This was done with reference to the cost standards and ratios of community home-based care service programs in typical Chinese provinces and cities, to provide an evidence-based basis for Chinese policymakers to optimize government subsidies and resource allocation.

## Methods

### Study design and study population

**Fig. 1:**
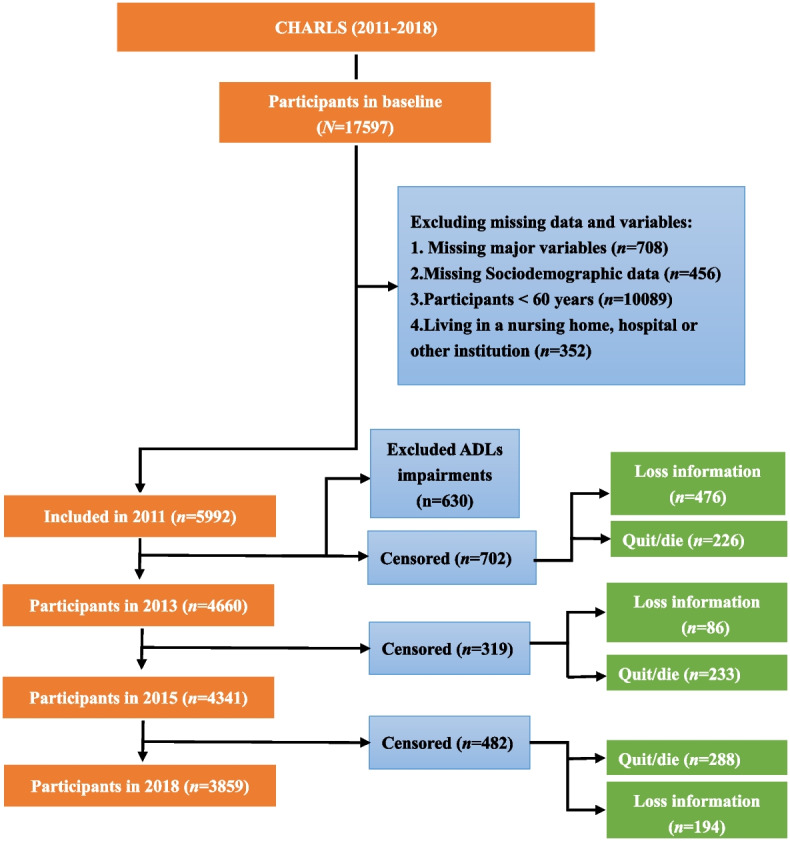
Sample screening flow chart

The data used in this paper were obtained from CHARLS database and the United Nations Population Division’s projections of the total older population by sex and age groups in China. CHARLS covers 28 provinces and has three characteristics: national, comprehensive and continuous, which can effectively reflect the dynamic changes of China’s older population. Details on the methods and sampling have been published previously [26]. The inclusion criteria for this study were older individuals aged 60 and above who resided at home or in the community. Based on a sample of 17,597 valid respondents from the 2011 CHARLS survey, the total number of older adults receiving community home-based care services was 7,156. After data preprocessing, we found that 1,164 study variables were missing or had incomplete key information, with missing rates ranging from 25 to 89%, particularly for blood biochemical indicators, with 535 missing values (45.96%). We used multiple imputation methods to fill in the remaining missing data in the study population and merged the means of multiple imputations. Finally, 5,992 eligible older individuals were included in the study. The sample screening flow chart is shown in Fig. 1. The study used CHARLS data to classify the multi-dimensional functional decline of older adults and measured the transfer probability matrix, combined with the United Nations Population Division data, to estimate parameters such as the scale of the migrating population by age and gender [27]. Age shift equation and survival backpropagation were used to correct for underreporting and misreporting. The analytical software used includes Stata 16.0, R version 4.0.2, SPSS 20.0 and Mplus 8.0.

### The core concept of eldercare definition

(1) Community home-based care: The concept of “community home-based care” was first proposed by the United Nations in the 1980s [28]. Its core concept is “a social care system for older adults that combines home-based care with community care”. It is widely defined as “a way of providing social care services to older adults at home, with the family as the core, the community as the support, and specialized service organizations as the carrier, combining both home-based and community-based services through the integration of formal and informal service resources in the community“ [29]. Specific service forms mainly include a service system consisting of social services provided by formal organizations and care, support, and care provided by informal networks. Formal organizations include community committees, government public departments, non-profit social organizations, and market-oriented enterprises that provide social services. Informal support networks mainly refer to social relationship networks of individuals such as family, relatives, and friends. This service system consists of three different service levels, namely basic care services, safety-net care services, and upgraded care services, with the former addressing basic care needs and the latter two addressing differentiated care needs [30].

(2) Long-term care for older adults: In 2016, the WHO unified various translations such as “long-term care”, “long-term nursing”, “long-term care”, and “long-term care for elderly” into “long-term care for older adults”. The National Center on Aging in the United States defines long-term care for older adults as a variety of services that meet the short-term or long-term health or personal care needs of older adults, improve and maintain their functional abilities, and promote individuals who cannot independently complete daily activities to live independently and safely as soon as possible. The care cycle can last for several weeks, months, or even years. Among them, continuous long-term care refers to the continuous help needed in daily life due to chronic diseases, permanent disabilities, dementia, and other reasons [31]. This article adopts the globally recognized concept of long-term care for older adults, which refers to systematic activities carried out by informal caregivers (including family, friends, or neighbors) and professional caregivers (health, social, and others) to ensure that older adults who lack self-care ability can maintain the highest quality of life according to their own priorities and enjoy the greatest possible independence, autonomy, participation, personal satisfaction, and human dignity. The long-term care system can maintain and improve the functional performance of disabled older adults and can be divided into formal and informal care categories [32].

(3) Formal care: Known as professional care, it includes all nursing and care services that are within the scope of formal employment regulations. It is a specialized care service provided by institutions and communities for older adults, mainly for those with severe disabilities, and provides training and support to family members such as children in professional care [33].

(4) Informal care: Known as family care or home care, it is unpaid care provided by people who have social relationships with the care recipient, such as spouses and children. It is based on commitment, love, and responsibility and has not become a commodity sold in the market system. Older adults with mild to moderate disabilities rely more on informal care provided by family members when they can choose their own care mode [34].

### Multi-dimensional functional decline related variables

The CHARLS data information used in analysis mainly covers issues on sociodemographic characteristics, family, behavior, cognitive, psychological, environmental and biological factors that affect health and longevity.

1. Sociodemographic characteristics: age, sex, place of residence, living environment, and educational levels.
2. Family factors: marital status, annual per capita household income, number of children, and residence with children or spouse.
3. Lifestyle behaviors: whether to participate in social activities, whether to drink alcohol, whether to smoke, and exercise ability.
4. Health behaviors: chronic illness, frailty, basic/instrumental activities of daily living ability, sleep time, self-rated health, life satisfaction, falls, managing illness, treatment status, use of assistive devices, formal/informal care status.
5. Biological factors: blood pressure, Body Mass Index (BMI).
6. Cognitive-psychological factors: cognitive ability, communication ability, memory, visual acuity, hearing, and mood.

### Multi-dimensional functional decline evaluation

Currently, most scholars use activities of daily living (ADLs) or instrumental activities of daily living (IADLs) as the basis for assessing disability statuses [35]. However, ADLs or IADLs can only reflect the serious functional impairment status of older adults by a single dimension. Studies on FC assessment criteria have found that most people have multi-dimensional FD [36], and the incidence of FD varies depending on the dimension and intensity of the FC examined [37]. Dysfunction is intricately linked to physical state, accessible resources, and environmental circumstances within the framework of “practical capability” [38]. Therefore, based on our previous research, this paper provides a comprehensive assessment of the multi-dimensional FC statuses for older adults in three dimensions: physical condition, care resource, and social interaction, which included self-care ability, motor ability, disease management ability, cognitive-psychological and communication skills, disease status, care resources, home environment, and social interaction ability, with a total of 29 indicators [39]. Combined with previously constructed dimensions and indexes, we used latent class analysis (LCA) method to classify the functional ability of older adults into categories for the years 2011,2013,2015, and the basic process of model construction for LCA is to first establish the baseline model (the number of potential categories is 1): that is, assuming complete independence among the exogenous variables, and using Maximum Likelihood Method to estimate each model, calculate the conditional probability and the response vector of the response vector. The models were then selected based on the AIC, BIC (the smaller the better fit), entropy value (greater than 0.8 is considered superior model fit) [40]. We used the Bootstrap Likelihood Ratio Test (BLRT) and Lo-Mendell-Rubin adjusted (LMR-A) test to compare the n-class model with the n-1 class model, where the smaller class is considered more optimal. The categories of functional abilities of older adults were named according to the unique conditional probabilities of multi-dimensional indicators in each class [41].

### Model construction

#### Multi-dimensional FC statuses transfer model construction

**Fig. 2:**
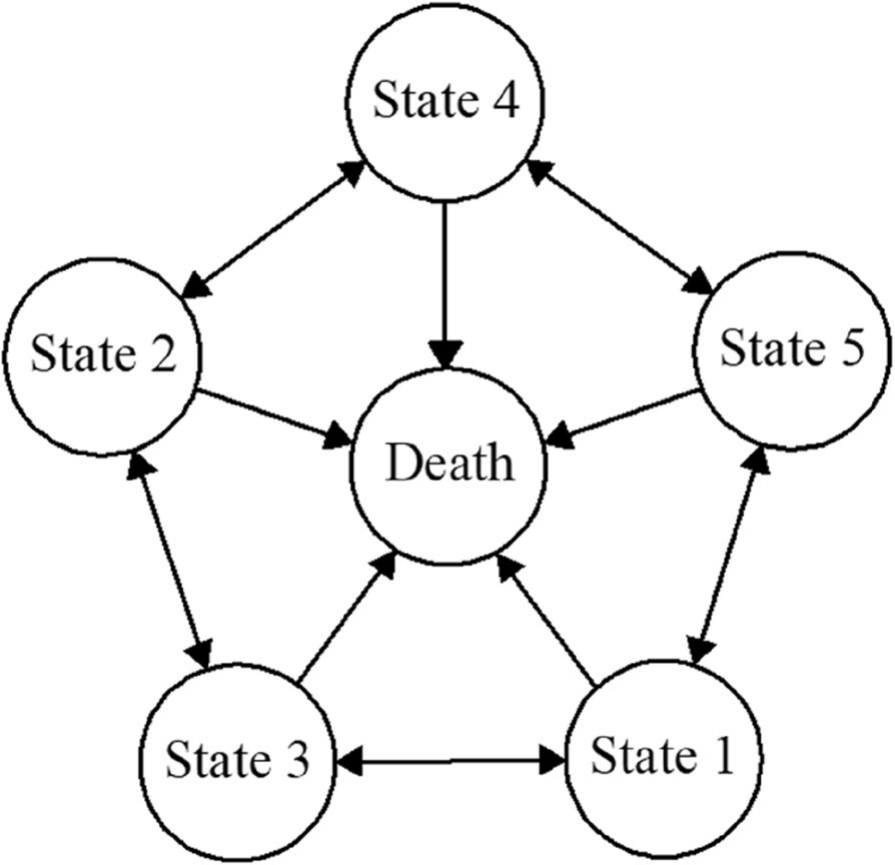
The transfer of various functional abilities of older adults. *Note*. state 1: viability disorder; state 2: acute disease; state 3: somatic functional disorder; state 4: health; state 5: sub-disorder status; state 6: death

Analyses were conducted by using the “msm (multi-state modeling)” R packages. By constructing a multi-state Markov model combining with CHARLS data, we use Markov transfer matrix to measure the multi-dimensional FC states transition probability of the older adults, and derive different states durations and population sizes in different periods based on actuarial theory. In a continuous-time multi-state Markov model, direct transitions between non-adjacent states are not allowed. This is consistent with the actual situation where there are hierarchical and dependency relationships between adjacent health states, that is, the transition between each functional state should show a stepwise, gradual change. Therefore, this model is suitable for calculating the transition between different levels of functional impairment, from state 1 to state 5 and death [42]. Based on this, a stochastic process model is set up to measure the transfer probability matrix of each FC states including states1, 2, 3, 4, 5, and 6, the first 5 states can achieve two-way transfer, while the death state is the absorbing state. Figure 2 shows the transformation diagram of the model.

In order to gauge the extent of demand for community home-based care, it is imperative to take into account the following crucial factors: first, the proportion of the older population with different FC states in China at the current stage; second, each FC status transfer probability and its change trend during the forecast period; third, the base period data of the older population in China during the forecast period. In this paper, we will study the above issues, measure the scale of the population with different degrees of FC from 2015 to 2035, and assess the scale of demand for community home-based care services.

1. Population gender and age structure are important factors affecting the prediction results. We used 2011 CHARLS data to calculate the proportion of the older population of different sexes and ages by FC statuses ${\beta }_{k,i}^{\alpha }$, where *i* = 1, 2, 3, 4, 5, and 6 represent five FC statuses and death respectively; *k* represents different age groups;α = 1,2 represent male or female, respectively. By using the hidden Markov Chain to forecast the transition probability from 2011 to 2013 and 2015 respectively, the probability transition situation of the next 5, 10, 15 and 20 years is predicted with 2015 as the starting point. This method ensures the span of the prediction interval is freely adjusted [43]. Based on the projection data of the United Nations Population Division [27], the population of older people of different sexes in each age group was multiplied with the corresponding vector of the proportion of older people in FC statuses ${\beta }_{k,i}^{\alpha }$to obtain each period population of different sexes and ages in each FC statuses.
2. The age group distance of this study was set at 10 years. There are three main considering reasons: (i) the same population cohort experienced similar period background, and the same chronological age group (e.g., 60–70 years old, 70 and above) can better reflect the mechanism and regularity of group FC state changes of external environmental factors such as economic and social development, i.e., the cohort effect; (ii) a small age group (e.g., divided according to 5 years old) can easily lead to male or female a small sub-sample size of a certain age group and a certain FC statuses causes low accuracy of state transfer probability estimation, which affects the prediction accuracy and stability; (iii) Referring to the experience of Zhang Zengxin (2019) who divided the disabled elderly according to the 10-year age group distance, the physical health statuses of the older people within the same age group has roughly similar distribution characteristics [44]. In order to coordinate the age group distance and transfer probability time, this paper calculated P (10) = e^10Q^= ${e}^{\frac{10lnP\left(3\right)}{3}}$ ,by matrix index method to improve the prediction accuracy.
3. To project the size of the older population by gender and age. The proportion of the older population in each FC state for each age group from 60 to 70 years old of different genders in 2011 was taken as the initial FC state distribution of the older population newly entering the age of 60 to 70 years old in the 10-year, 20-year, and 30-year periods. At the same time, the vectors of each FC state and the older population in different sexes and age groups 2011 were projected by the probability matrix of FC state transfer to obtain the population size distribution of the older population in different states over time in the period 2015–2035; the proportion of each FC state for each age group of 60–70 years for both sexes was multiplied with the vector of new entry into the 60–70 age in different genders group in each 10-year period. At the end to obtain of population size of each FC statuses of older people aged 60–70 in different genders in 2020–2035.
4. In addition, in 2011, the Ministry of Civil Affairs of the People’s Republic of China released the “12th Five-Year Plan for the Construction of Social Aged Care Service System”, which further advocates the new “90–7 – 3” policy for the aged care, that is, 90% of the aged care with the assistance of socialized services through family In other words, 90% of the elderly will be taken care of at home with the assistance of social services, 7% of the elderly will be taken care of by the government through community care services, and 3% of the elderly will be taken care of in the eldercare service institutions. Based on this, the scale of labor demand in China’s elderly home-based care market under the “90-7-3” elderly care pattern is estimated based on the “97%” social and family care ratio.

### Cost projections for community home-based care services for the older adults

There are certain differences in the service content, service time and service level required by the older population with different levels of FD, and it is obviously unreasonable to use the same consumption standard. To determine the total cost of eldercare services, we utilized both project cost analysis and man-hour costing methods based on different parameters for eldercare service levels. The man-hour costing method mainly focuses on labor value and factors in changes in family caregiver opportunity costs, economic growth rates, and wage levels in relation to population size. In contrast, project cost analysis focuses on the specific costs of each service project, which are primarily related to service difficulty levels. It is important to consider the cost information and differences reflected by both methods when predicting future long-term care costs for improving the accuracy of cost predictions.

1. The project cost method: by using the unit price of each service, combining the frequency of different care levels of service demand, the cost of community home-based care included in different years is forecasted. Based on a preliminary literature review, community home-based care service content has been divided into daily life care, basic care, professional medical care services, auxiliary monitoring, spiritual comfort, medical care and education and training. As Shanghai and Beijing have a more severe levels of aging and have started piloting long-term care systems earlier. This study used the project cost method to measure the cost of community home-based care, based on the statistical information disclosed by the Shanghai and Beijing government [45]. A preliminary framework for cost accounting was formed after several experts’ discussions, the cost was mainly based on the cost of family care at the household level, nursing care services provided by professional caregivers. The accounting method for the project cost of long-term care services was determined as follows: project cost = direct cost (labor cost + material cost + transportation cost) + indirect cost (management cost + education cost). The project cost calculation formula:
  1. material costs (A) = disposable material costs (A1) + less than 500 yuan of equipment and instruments and low-cost consumables (A2) = the unit price of the relevant materials used in a single care operation (including tax) * actual consumption + (item unit price / expected number of recyclable) * the number of single project operations used.
  2. manpower cost (B) = single care hours (B1) * unit time manpower cost (B2) * number of project operations = average monthly wage / average monthly hours * (direct care hours + indirect care hours) * number of project implementation.
  3. Transportation cost (C) = (door-to-door transportation time x unit time manpower cost) + door-to-door transportation cost + depreciation cost of transportation.
  4. Management cost (D)=(A + B + C)*5%.
  5. Education cost (E)=(A + B + C + D)*5%.
    The total cost of community home-based care is equal to the sum of the above five items.
2. Man-hour costing method: Based on this paper’s classification of FD levels for older adults, we combined with the Shanghai community home-based care service grading standards and service duration of different grades and scholar Cao Xinbang’s measurement [46]. The care modalities for home-care are divided into 3 levels of care according to the average care hours required from low to high: 1.5 and 4.5 h of service per week for assessment states 2 and 5, 7.42 h of service per week for assessment state 3, and 10.58 h for state 1. According to the requirements of the national health department, the ratio of community home-based care cost and the corresponding cost of designated medical care institutions is 1.8. Labor cost accounts for a large proportion of the care cost, and the prediction of labor cost in this study is based on the average wage income of employed persons in urban units as an indicator. Statistics released by China’s Bureau of Statistics in recent years show that the average wage of employed persons in urban non-private units in China in 2020 is 97,379 yuan, and the average wage income per person per hour is 48.50 yuan (measured based on 251 days and 8 h of work per person per year and day, respectively). According to the growth rate of China’s economic development, assuming that the economic growth rate is synchronized with the level of residents’ wage income, and referring to the prediction results of Li Shantong equivalent (2003) country on China’s economic growth rate, the average annual economic growth rate is set at 6.5% from 2010 to 2020; 5.4% from 2020 to 2030; and 4.5% from 2030 to 2040 [47]. By using the average annual economic growth rate as the trend of care service price changes in a specific year, we forecast the total cost of eldercare services by different genders and ages of home-care groups in 2020–2035, combined with relevant parameters such as the size of disabled older population and the price of care services during the projection period. The formula for calculating the cost of community home-based care is as follows [48]:

Monthly benefit cost of home/community care = care time required for each FD level for one week × 4 (indicating 4 weeks per month) × cost coefficient of community home-based care (1.80) × average hourly wage of employed workers (48.50 yuan).

## Results

### The characteristics and sensitivity analysis of study participants and excluded sample

A sensitivity analysis between excluded sample with missing values and study participants was conducted to reduce information bias and demonstrate the representativeness and accuracy of participant selection. A differential analysis of the basic epidemiological characteristics and blood biochemical indicators of the two groups was conducted. The results showed that the age of the study population and excluded sample were 68.08 ± 6.67 and 69.31 ± 7.88, respectively, and males accounted for 49.97% and 50.99%, respectively, which were relatively close. Apart from variations in place of residence, caregiving time, hemoglobin, and triglycerides (within 5% ), there were no statistically significant differences in other indicators. This suggests that the two groups were generally comparable, and the sample used in this study was representative and produced reliable results. By conducting a sensitivity analysis, we were able to provide additional evidence of the robustness and validity of our findings, which enhances the scientific rigor of our study (Tables 1, 2 and 3).

**Table 1:** Sensitivity analysis: basic epidemiological characteristics of study population and excluded sample

Category	General information	Variables	Study population (‾*x* ± *s*/%)	Excluded sample (*x* ± *s*/%)	*P* value
	**Gender**	Men	2994 (49.97)	593 (50.99)	0.519
		Women	2998 (50.03)	570 (49.01)	
		Missing value		0	
	**Age (years)**	60 ~ 69	3559(59.40)	623(53.57)	0.945
		≥ 70	2433(40.60)	540(46.43)	
		Missing value		0	
	**Residential areas**	Urban areas	1226 (20.46)	169 (14.57)	0.035
		Rural areas	4766 (79.54)	991 (85.43)	
		Missing value		4 (0.26)	
	**BMI (kg/m** ^=**2**^ **)**	BMI index	22.88 ± 4.12	22.35 ± 4.20	0.714
		Missing value		1009 (86.68)	
	**Blood pressure (mmHg)**	Systolic pressure	136.03 ± 25.37	136.85 ± 24.05	0.507
		Diastolic pressure	75.21 ± 11.81	74.69 ± 11.75	0.600
		Missing value		1014 (87.11)	
	**Educational level**	Illiterate	2212 (36.92)	430 (37.20)	0.538
		Primary school	2721 (45.41)	444 (38.41)	
		Vocational school	951 (15.87)	243 (21.02)	
		College and above	108 (1.80)	39 (3.37)	
		Missing value		8 (0.69)	
	**Marital status**	Married	4705 (78.52)	907 (77.99)	0.679
		Divorced	1238 (20.66)	243 (20.89)	
		Single	49 (0.82)	13 (1.18)	
		Missing value		1 (0.09)	
**Social behavior**	**Sleep duration (h)**	Sleep time	6.17 ± 2.02	6.84 ± 2.32	< 0.001
		Missing value		366 (31.44)	
	**Smoking**	Yes	2552 (42.59)	476 (40.96)	0.253
		No	3440 (57.41)	686 (59.04)	
		Missing value		2 (0.17)	
	**Alcohol consumption**	≥ 1time/month	1404 (23.43)	269 (23.19)	0.702
		<1time/month	398 (6.64)	82 (7.07)	
		No alcohol consumption	4190 (69.93)	809 (67.74)	
		Missing value		4 (0.26)	
	**Life satisfaction**	Extremely satisfied	99 (1.65)	18 (2.69)	0.831
		Very satisfied	1208 (20.16)	140 (20.96)	
		Fairly satisfied	3940 (65.75)	435 (65.12)	
		Not very satisfied	613 (10.23)	64 (9.58)	
		Not at all satisfied	132 (2.21)	11 (16.47)	
		Missing value		496 (42.61)	
	**Self-rated health**	Very good	206 (3.44)	27 (4.62)	0.300
		Good	750 (12.52)	99 (16.92)	
		Fair	2675 (44.64)	221 (37.78)	
		Poor	1831 (30.56)	193 (32.99)	
		Very poor	530 (8.84)	45 (7.69)	
		Missing value		579 (49.74)	
	**Number of children**	0	1662 (27.74)	543 (47.47)	0.416
		1 ~ 2	3581 (59.76)	201 (17.57)	
		3 or more	749 (12.5)	400 (34.97)	
		Missing value		20 (1.72)	
	**Long-term care (h)**	Formal care	19.84 ± 72.93	22.90 ± 90.52	< 0.001
		Informal care	7.24 ± 40.96	4.46 ± 101.16	< 0.001
		Missing value		915 (78.61)	

**Table 2:** Codes of blood biochemical indexes

Category	Variable	Abbreviation	Units
Inflammatory Response Indicators	White Blood Cell Count	WBC	10^3^
	Platelet Count	PLT	10^9^/L
	C-reactive Protein	CRP	mg/L
Blood Traits	Packed Cell Volume	PCV	%
	Mean Corpuscular Volume	MCV	fL
	Hemoglobin	HGB	g/dL
Renal Function Indicators	Blood Urea Nitrogen	BUN	mg/dL
	Blood Creatinine	CREA	mg/dL
	Cystatin C	CysC	mg/L
	Uric Acid	UA	mg/dL
Blood Lipid Levels	Total Cholesterol	TC	mg/dL
	Triglycerides	TG	mg/dL
	High-Density Lipoprotein Cholesterol	HDLC	mg/dL
	Low-Density Lipoprotein Cholesterol	LDLC	mg/dL
Blood Glucose Levels	Glycated Hemoglobin	HbAlc	%
	Glucose	GLU	mg/dL

**Table 3:** Sensitivity analysis: characteristics of blood biochemical indexes of the study population and excluded sample

Name	Category	Biochemical Indicators	Study population [‾*x* ± *s/* (*n* = 5992)]	Excluded sample [‾*x* ± *s/* (*n* = 629)]	*P* value
**Blood Biochemical Indicators**	Inflammatory Response Indicators	WBC (10^3^)	6.27 ± 1.93	6.32 ± 1.96	0.508
		PLT (10^9^/L)	207.11 ± 73.46	203.44 ± 78.14	0.354
		CRP (mg/L)	3.32 ± 8.65	3.78 ± 9.58	0.218
	Blood Traits	PCV (%)	41.11 ± 6.37	41.41 ± 5.69	0.275
		MCV (fL)	91.36 ± 8.50	90.99 ± 8.17	0.316
		HGB (g/dL)	14.24 ± 2.18	14.46 ± 2.30	0.021
	Renal Function Indicators	BUN (mg/dL)	16.38 ± 4.78	16.78 ± 5.79	0.098
		CREA (mg/dL)	0.82 ± 0.29	0.82 ± 0.33	0.861
		CysC (mg/L)	1.12 ± 0.30	1.13 ± 0.33	0.496
		UA (mg/dL)	4.6 ± 1.30	4.60 ± 1.35	0.928
	Blood Lipid Levels	TC (mg/dL)	194.63 ± 38.65	192.96 ± 39.38	0.312
		TG (mg/dL)	127.94 ± 95.05	140.10 ± 122.90	0.018
		HDLC (mg/dL)	51.75 ± 15.54	50.89 ± 15.92	0.197
		LDLC (mg/dL)	118.00 ± 34.99	116.36 ± 35.99	0.278
	Blood Glucose Levels	HbAlc (%)	5.31 ± 0.82	5.35 ± 1.03	0.286
		GLU (mg/dL)	112.47 ± 39.52	111.58 ± 35.65	0.594

### Classification and definition of FC

After specifying the content of functional capacity indicators for older adults, we further categorized them. Table 4 shows the indicator fits for the LCA of the seven functional capacity conditions. Both AIC and BIC decreased when the number of potential categories went up from 2 to 7, and BIC and AIC reached the lowest when the potential category was 7. However, the entropy values were greater than 0.8 within 5 categories and less, which indicated that the explanatory strength of the model was greater than 80%. The test level for both Bootstrap Likelihood Ratio Test (BLRT) and Lo-Mendell-Rubin adjusted (LMR-A) test was set at 0.05. Our results showed that the *P*-values for both tests are less than 0.05, indicating that the model fit of the five categories is better than the model of the four categories. Therefore, combining the actual situation and the results of data analysis, the model with the number of categories of 5 was selected to have the best fit for the results. We further applied LCA to classify the functional ability of older adults into categories for the years 2013,2015 follow-up period to validate the stability and scientific validity of the classification by using (Supplementary Tables S1 and S2).

**Table 4:** Latent class model and fit indices

Model	K	AIC	BIC	SSA-BIC	Entropy	LMR-A *p*-value	BLRT *p*-value	Classification probability
1	42	207786.964	208068.29	207934.823				1
2	85	196672.913	197242.259	196972.152	0.812	< 0.001	< 0.001	0.62/0.38
3	128	194977.932	195835.3	195428.551	0.862	< 0.001	< 0.001	0.10/0.31/0.58
4	171	192837.12	193982.509	193439.119	0.805	< 0.001	< 0.001	0.12/0.15/0.33/0.39
5	214	192196.131	193629.541	192949.509	0.823	< 0.001	< 0.001	0.11./0.10/0.77/0.34/0.37
6	257	191593.208	193314.64	192497.966	0.776	0.378	< 0.001	0.08/0.07/0.27/0.33/0.10/0.15
7	300	191223.427	193232.881	192279.565	0.735	0.7812	< 0.001	0.08/0.10/0.20/0.19/0.22/0.16/0.07

*BIC *Bayesian Information Criterion, *SSA-BIC *Sample-Size Adjusted BIC, *AIC *Akaike Information Criterion, *LMRA-A *Lo-Mendell-Rubin adjusted likelihood ratio test, *BLRT B*ootstrap likelihood ratio test

**Fig. 3:**
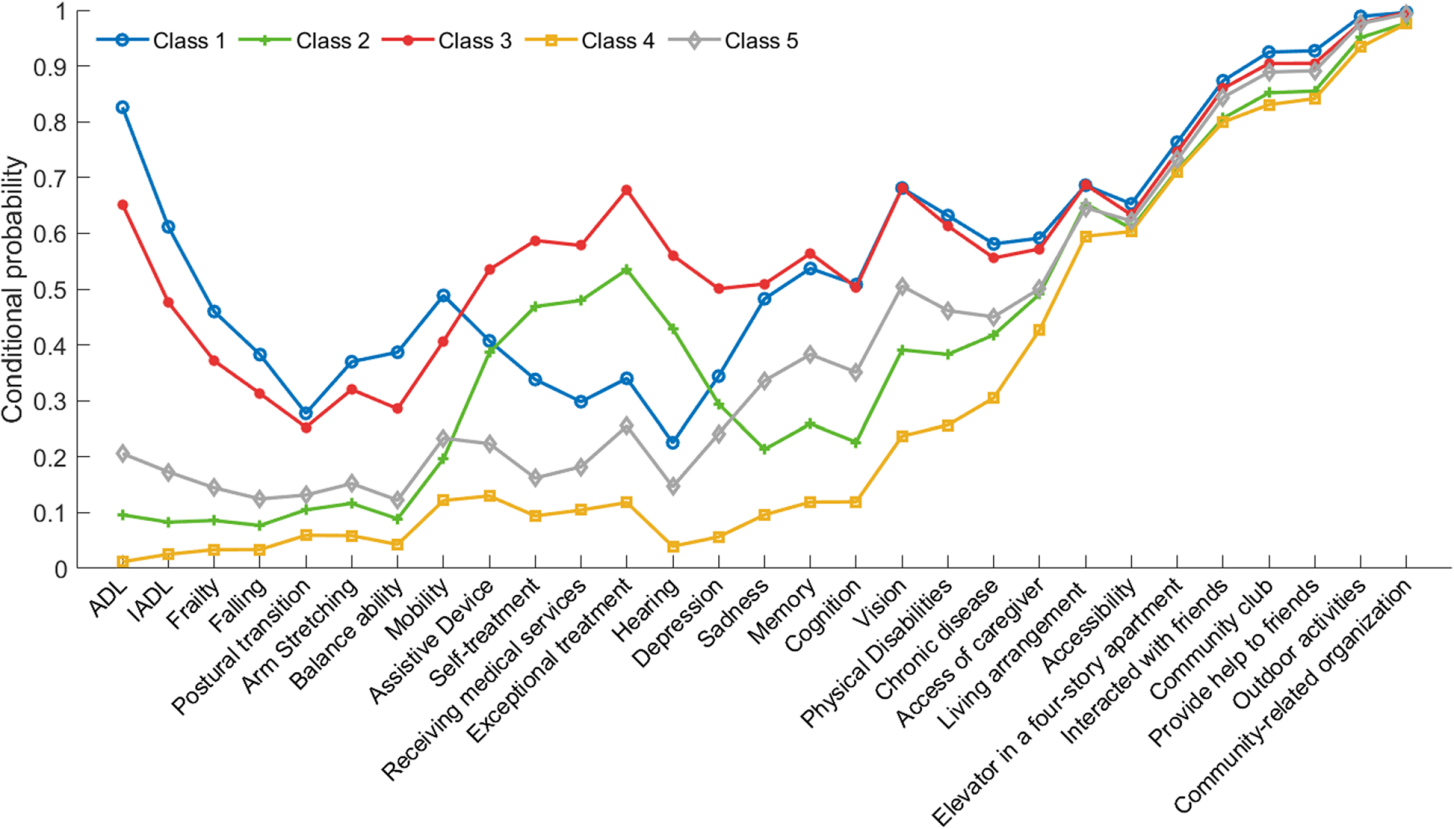
Multi-dimensional disability indexes of physical condition, care resources and social interaction by class. *Note.* state 1: viability disorder; state 2: acute disease; state 3: somatic functional disorder; state 4: health; state 5: sub-disorder status; state 6: death

Figure 3 indicated the distribution of the conditional probabilities of different potential categories. State 1 was defined as “viability disorder” because of the poor ability of daily living; State 2 had a higher probability of using aids, seeking medical treatment, and special treatment, while other aspects of functioning were in good condition, which was defined as “acute disease”; In state 3, there was a higher conditional probability of being poor in all aspects of functioning (i.e., higher conditional probability), especially in medical treatment and use of assistive devices. And the conditional probability of all indicators in this category is higher than that in the other four categories, thus defining this group as “somatic functional disorder “; State 4 had a lower probability of functional disorder than the other 4 potential categories, hence this population was defined as “health”; State 5 had a lower conditional probability of disease status than state 2, but a higher conditional probability of overall functional status than state 2 and 4, and was in the transition stage from health to functional impairment. This population was thus defined as “sub-disorder status”.

This has been described in detail in our previous studies, which can be referred to the published articles [49]. The categories “viability disorder”, “acute disease”, “somatic functional disorder”, and “sub-disorder status” belong to FD, which were in certain impairments in FC and need eldercare. Among them those in “sub-disorder status” or “acute disease” states might need some cycles of rehabilitation physical therapy services. And those in states “viability disorder” and “somatic functional disorder” were closed to the disability status, who need more professional nursing. (See Supplementary Tables S3 for classification details)

### Descriptive analysis of FC categories of older adults

The composition of the different genders disabling conditions in 2011 was shown in supplementary Table S2-S3. For male, the proportion of FC of each group accounted for the total sample was as follows, 8.4% for the category “viability disorder”, 10.6% for “acute diseases”, 5.8% for “somatic functional disorder”, 41.9% for “Health”, 33.0% for “sub-disorder status”. The proportion of the female sample was: the category “viability disorder” accounted for 14.3%, “acute diseases” accounted for 10.0%, “somatic functional disorder” accounted for 9.6%, 25.9% for “Health”, and 40.0% for “sub-disorder status”. All the above categories except “health” belong to FD.

### The FC states transition probability matrix

Table 5 summarizes the transition probability matrix for each state over a one-year period and explains the specific meaning of the transition values. It was found that for older adults in the baseline state, the probability of transitioning from state 1 to state 3 was higher than that of transitioning to state 4. An analysis of the estimated intensity matrix for male older adults revealed that the likelihood of transitioning from state 3 to state 1 was approximately 23.01% higher than that of transitioning from state 2 to state 1. The transitions from state 3 to state 1 were 22.14 and 8.61 times more likely for those in state 4 and state 5, respectively. The estimated intensity matrix for female older adults indicated that the probability of transitioning from state 3 to state 1 was similar to that of transitioning from state 2 to state 1. The transitions from state 3 to state 1 were 10.56 and 4.83 times more likely for those in state 4 and state 5, respectively. A feature analysis of the transition probability matrix revealed that the transition patterns were similar across different age groups but varied by gender. Taking female older adults aged 60–69 in the baseline state as an example, the results showed that individuals in state 1 had a 38.9% probability of remaining in that state in the first year. In the transitional state, they were most likely to progress to state 3. For older adults in state 2, the probability of maintaining their original state was only 27.4%, and the likelihood of transitioning to state 3 and state 5 was higher than that of transitioning to state 1 (35.0%, 16.7% vs. 16.5%, respectively). For older adults in state 5, the probability of remaining at that level was the highest (57.6%), and the probability of transitioning to state 1 was lower than that of transitioning to state 3 (1.8% vs. 7.4%). For older adults in state 4, the likelihood of transitioning to state 5 was 34.9 times higher than that of transitioning to state 1. For male older adults in the baseline state aged 60–69, the probability of remaining in state 1 in the first year was 27.8%, and they were most likely to progress to state 3 in the transitional state. For older adults in state 2, the probability of maintaining their original state was only 21.7%, and the likelihood of transitioning to state 3 and state 5 was higher than that of transitioning to state 1 (32.0%, 25.9% vs. 12.6%, respectively). For older adults in state 5, the probability of remaining at that level was the highest (68.3%), and the probability of transitioning to state 1 was lower than that of transitioning to state 3 (3.5% vs. 12.5%). Those in state 4, the likelihood of transitioning to state 5 was 85.2 times higher than that of transitioning to state 1. Among older adults in the five functional states at baseline, female older adults aged 60–69 in states 2 and 3 had the highest probability of death, followed by states 1, 4, and 5. Female older adults aged 70 or older had the highest probability of death in state 1. Male older adults aged 60–69 in state 3 had the highest probability of death, followed by states 2, 1, 4, and 5. Female older adults aged 70 or older in state 3 had the highest probability of death, followed by states 1, 2, 5, and 4.

The results of the transition risk ratio analysis between different functional states, genders, and ages showed that females [HR 0.72 (95%CI: 0.60–0.86)] and those aged 70 or above [0.42 (95%CI: 0.35–0.51)] had a reduced probability of transitioning from state 2 to state 5, indicating that older women with functional impairments had difficulty recovering. Similarly, females [1.56 (95%CI: 1.39–1.76)] and those aged 70 or older [1.51 (95%CI: 1.35–1.70)] had an increased risk of transitioning from state 5 to state 2, indicating that older women were more likely to experience acute illnesses and had difficulty transitioning from sub-disorder functional status to healthy states. Furthermore, those aged 70 or older [6.89 (95%CI: 1.38–35.42)] had an increased risk of transitioning from state 2 to death.

**Table 5:** Transformation intensity and one-year transition probability of Markov model

Age (year)	States	Man						Women					
		State 1	State 2	State 3	State 4	State 5	State 6	State 1	State 2	State 3	State 4	State 5	State 6
**60 ~ 69**	State 1	0.278	0.212	0.360	0.010	0.107	0.033	0.389	0.223	0.291	0.010	0.077	0.010
	State 2	0.126	0.217	0.320	0.037	0.259	0.040	0.165	0.274	0.350	0.029	0.167	0.015
	State 3	0.155	0.231	0.349	0.022	0.190	0.053	0.169	0.276	0.352	0.027	0.162	0.015
	State 4	0.007	0.044	0.038	0.305	0.599	0.007	0.016	0.063	0.075	0.282	0.559	0.006
	State 5	0.018	0.073	0.074	0.142	0.683	0.010	0.035	0.102	0.125	0.157	0.576	0.004
	State 6	0.000	0.000	0.000	0.000	0.000	1.000	0.000	0.000	0.000	0.000	0.000	1.000
**≥ 70**	State 1	0.211	0.327	0.283	0.007	0.075	0.097	0.290	0.334	0.252	0.006	0.049	0.068
	State 2	0.159	0.335	0.274	0.017	0.131	0.084	0.201	0.373	0.279	0.013	0.083	0.052
	State 3	0.168	0.335	0.277	0.012	0.109	0.099	0.202	0.373	0.279	0.013	0.081	0.052
	State 4	0.015	0.077	0.046	0.293	0.544	0.025	0.031	0.110	0.080	0.260	0.484	0.035
	State 5	0.037	0.134	0.090	0.121	0.598	0.020	0.065	0.183	0.134	0.127	0.471	0.019
	State 6	0.000	0.000	0.000	0.000	0.000	1.000	0.000	0.000	0.000	0.000	0.080	1.000

State 1: viability disorder; state 2: acute disease; state 3: somatic functional disorder; state 4: health; state 5: sub-disorder status; state 6: death

Table 6 displays the 5-year transition probability matrix for functional capacity statuses of older individuals by gender and age group. Supplementary Tables S4-S6 present the transition probability matrices for the 10-year, 15-year, and 20-year periods. Based on the overall analysis, the likelihood of functional dependency and mortality for older adults of both genders, irrespective of their initial health status, tends to progressively increase with advancing age. Particularly, older adults in state 4 exhibit the best health status, but have a lower probability of transitioning to other states compared to other categories, for both males and females. Conversely, individuals in state 5 are in a transitional phase between good health and functional dependency, and have the highest probability of transitioning to other states across time. As a result, individuals in state 5 are more likely to experience both improvements and deteriorations in health, and have the potential to transition from states 1, 2, 3, and 4. Furthermore, as individuals age and their initial health status deteriorates, the probability of transitioning to state 2 increases, with minimal gender differences. The likelihood of transitioning to state 3 increases incrementally with age for males, but decreases with age for females with overall poorer initial health status. Males have a lower probability of transitioning to state 1 compared to females, while the likelihood of transitioning to state 1 for females is much lower than the probability of transitioning to state 3.

**Table 6:** Probability matrix of 5-year FC states transfer by gender and age group

	Male						Female					
FC statuses	State 1	State 2	State 3	State 4	State 5	State 6	State 1	State 2	State 3	State 4	State 5	State 6
60–69 year												
State 1	0.085	0.129	0.183	0.083	0.369	0.151	0.154	0.197	0.250	0.073	0.273	0.054
State 2	0.077	0.122	0.170	0.092	0.395	0.144	0.145	0.191	0.242	0.078	0.286	0.058
State 3	0.079	0.122	0.172	0.087	0.379	0.162	0.146	0.191	0.242	0.078	0.286	0.058
State 4	0.059	0.189	0.143	0.122	0.496	0.070	0.118	0.171	0.216	0.104	0.355	0.036
State 5	0.062	0.111	0.148	0.118	0.481	0.080	0.123	0.175	0.222	0.099	0.343	0.037
State 6	0.000	0.000	0.000	0.000	0.000	1.000	0.000	0.000	0.000	0.000	0.000	1.000
≥ 70 year												
State 1	0.093	0.193	0.156	0.040	0.184	0.334	0.155	0.262	0.196	0.029	0.116	0.242
State 2	0.093	0.194	0.157	0.043	0.196	0.318	0.157	0.265	0.199	0.031	0.121	0.227
State 3	0.092	0.191	0.154	0.041	0.189	0.333	0.157	0.266	0.199	0.031	0.121	0.227
State 4	0.088	0.196	0.154	0.074	0.306	0.182	0.148	0.262	0.196	0.051	0.175	0.169
State 5	0.091	0.200	0.158	0.068	0.287	0.196	0.154	0.269	0.201	0.046	0.163	0.168
State 6	0.000	0.000	0.000	0.000	0.000	1.000	0.000	0.000	0.000	0.000	0.080	1.000

State 1: viability disorder; state 2: acute disease; state 3: somatic functional disorder; state 4: health; state 5: sub-disorder status; state 6: death

### Verification of the reliability of the FC statuses transfer probability matrix

#### Validation analysis of predicted transition probabilities for each state

In view of the effect of missing data on predictive models, Tables S4-6 comprised individuals with three or more follow-up records, whereas Tables S7-S10 consisted of those with at least two follow-up records. These two groups of participants were then incorporated into a Markov model to compute probability values for intervals of 5, 10, 15, and 20 years. The transition probabilities between the two groups were compared and analyzed. The maximum difference between the probability matrices of the two participant groups was only 1.1%, indicating that the predicted probability values were relatively stable. In addition, we calculated the 1-year transition probability matrix for the new model and estimated the transition probabilities for different years using numerical shooting. The maximum difference between the estimated probabilities was only 2.45%, demonstrating the consistency of the results and further verifying the accuracy and reliability of the predicted results.

#### Evaluation of Markov model

**Fig. 4:**
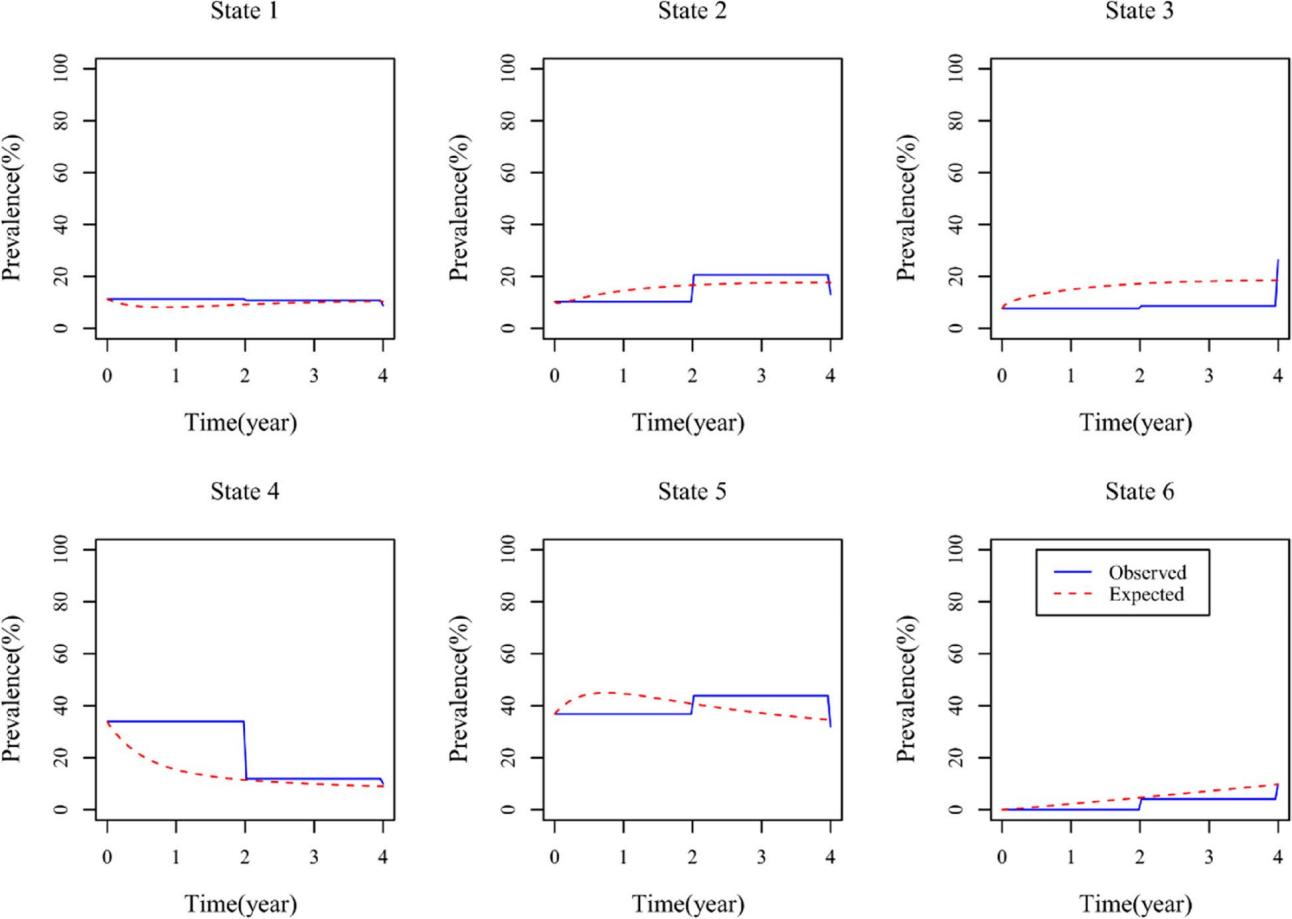
Fitting of the actual and expected conversion rates of the functional ability states of participants

To evaluate the fitting performance and efficacy of the model, we compared the actual observation and expected incidence rates of functional ability states over time (2011–2015), as shown in Fig. 4. We also constructed contingency tables for actual and expected transition probabilities, akin to the classical Pearson χ^2^ test. The results showed that the test statistic was *T* = 1165.89 and *P* = 0.14, indicating that the actual and expected incidence rate curves matched well at different time points, and the model fitting performance was good.

#### Sensitivity analysis of the model setting

We compared the results with an alternative model and performed a likelihood ratio test (LRT) to reach the predictive performance of different models. We constructed a new model that allowed for all possible transitions (direct and indirect) among state 1 and state 5, and compared it with the original model using the LRT. The results showed that when *df* = 5, the test statistic *G* was 4.18 and *P* = 0.32, indicating that there was no significant statistical difference between the original and new models, demonstrating the robustness and accuracy of the original model.

#### Validation analysis of predicted results

According to the World Population Prospects (2019 Revision) data and the FC statuses transfer probability measured by CHARLs from 2020 to 2035, we estimated the FC statuses distribution of the older population aged over 60 in China in 2020. It was estimated that the healthy older adults were about 20.985 million, and the older population with FD in states 1, 2, 3 and 5 were 31.915, 52.989, 53.408 and 82.986 million, respectively (Table S11). The average prediction error for the older population size across different genders, ages, and functional ability states was within 5%, indicating good prediction accuracy. The results also agreed with the China National Research Center on Aging predictions (2019 Revision) [27] and the projection results of the group of China Research Center on Aging [50].

### Prediction and analysis of the size of older population by gender, age and different FD states, 2020–2035

**Fig. 5:**
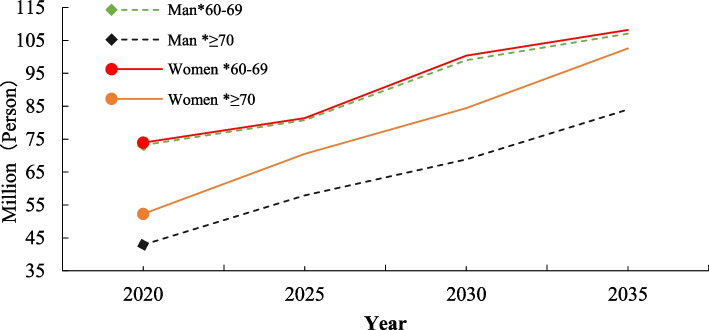
The changing trend of the projected size of older population in different gender and age group from 2020 to 2035(Million)

Based on the above prediction results, we further obtained the size of the older population in each FD statuses by gender and age group in the prediction period (2020–2035), which is shown in Table 7; Fig. 5 and supplementary Table S11.

1. Over the next 15 years, from 2020 to 2035, the total number of older adults is expected to increase from 242.283 million to 401.80 million, while the total population size in States 1, 2, and 3 is projected to rise from 138.313 million to 236.581 million by 2035. In the 60–69 and ≥ 70 age groups, the number of female older adults in States 1, 2, and 3 is higher than that of males. The total number of females in states 1, 2, and 3 is expected to increase to 144.37 million, representing a growth of approximately 172%, while the total number of males in States 1, 2, and 3 is anticipated to increase to 92.21 million, a rise of around 170% from 2020 to 2035.
2. The population size of older adults in State 4 is comparable for both genders across different age groups (60–69, ≥ 70). In contrast, the ratio of the number of older men to older women in State 5 is higher across different age groups, but overall, the increase in the number of older people from 2020 to 2035 is comparable, with an expansion of 1.60 times for men and 1.58 times for women.
3. The community home-based older population in State 6 is projected to increase from 47.549 million in 2020 to 88.036 million in 2035, accompanied by an increase in the death rate from 19.57% to 2020 to 21.91% in 2035. Moreover, the ratio of the number of older women to older men in the 60–69 age group in state 6 is higher, while overall, the expansion of the number of older people in state 6 in the 60–69 age group is much greater than that in the ≥ 70 age group.

**Table 7:** The projected size of the older population with different FC statuses by gender and age group (Million)

	Male				Female			
FC statuses	2020	2025	2030	2035	2020	2025	2030	2035
60–69								
State 1	5.504	6.168	7.575	8.187	10.579	11.711	14.449	15.575
State 2	9.387	10.448	12.810	13.857	14.310	15.803	19.498	21.013
State 3	12.716	14.190	17.409	18.828	18.132	20.021	24.707	26.624
State 4	8.847	9.682	11.852	12.817	6.779	7.410	9.136	9.844
State 5	36.727	40.296	49.340	53.365	24.133	26.432	32.588	35.120
State 6	9.217	14.428	19.095	23.263	20.806	23.39	25.849	28.192
≥ 70								
State 1	5.541	7.351	8.725	10.645	10.291	14.007	16.785	20.390
State 2	11.694	15.589	18.516	22.591	17.598	23.882	28.613	34.754
State 3	9.389	12.495	14.836	18.100	13.172	17.877	21.419	26.017
State 4	3.001	4.191	4.976	6.073	2.358	3.073	3.672	4.459
State 5	13.269	18.285	21.792	26.598	8.857	11.665	13.948	16.940
State 6	9.521	13.797	16.919	19.185	8.005	11.922	14.993	17.396

*FC *Functional capability; state 1: viability disorder; state 2: acute disease; state 3: somatic functional disorder; state 4: health; state 5: sub-disorder status; state 6:death

### Projected the cost of eldercare with different levels of FD

#### Total project cost analysis

Firstly, we classified the caregiving needs of older adults into six states based on the severity of functional impairment, ranging from mild to severe: state 4, state 5, state 2, state 3, and state 1, all of which ultimately transition to the death state (state 6). Since state 4 corresponds to the health status defined by WHO, as previously discussed, this group of people does not require community home-based eldercare and is thus excluded from the population in need of long-term care. To project the total service cost needs for older adults with functional dependency statuses of state 1, 2, 3, and 5, we utilized a Markov model and cost projection method, which are presented in Table 8. The details of service content and frequency coefficient for each service item among 4 categories are shown in Tables S12 and S13. The total costs of program from 2020 to 2035, are 1668.623 billion, 2036.910 billion, 2468.829 billion, 2836.754 billion yuan, separately. Table S14 presented that older adults in states 3 and 5 at age 60–69 spend 1-2.8 times care cost more than those over 70, except states 1 and 2. However, both in the 60–69 and the ≥ 70 age group, women spend 1.4–1.92 times more than men in each category except the fifth one.

**Table 8:** Projected cost analysis of eldercare cost for community home-based older individuals by gender and FD statuses from 2020 to 2035 (Unit: billion RMB)

Age (years)	States	Men				Women			
		2020	2025	2030	2035	2020	2025	2030	2035
**60 ~ 69**	State 1	109.950	123.216	151.314	163.539	211.312	233.926	288.622	311.121
	State 2	44.163	49.156	60.268	65.194	67.327	74.352	91.732	98.863
	State 3	178.340	199.017	244.171	264.073	254.313	280.805	346.516	373.406
	State 5	14.486	15.893	19.460	21.048	9.518	10.425	12.853	13.852
**≥ 70**	State 1	110.687	146.831	174.288	212.632	205.571	279.794	335.293	407.300
	State 2	55.017	73.345	87.114	106.285	82.797	112.362	134.618	163.513
	State 3	131.680	175.240	208.073	253.858	184.737	250.736	300.410	364.899
	State 5	5.233	7.212	8.595	10.491	3.493	4.601	5.501	6.681

*FD *Functional decline; state 1: viability disorder; state 2: acute disease; state 3: somatic functional disorder; state 5: sub-disorder status

Table 9 presents the service cost trends for community home-based older individuals, which are expected to gradually increase from 2020 to 2035 by approximately 100–180%. Notably, there are significant cost variations among different functional care states and service categories. For instance, in 2035, as shown in Tables S15 and S16, the total costs for care states 1, 2, 3, and 5 were 1094.591 billion, 433.855 billion, 1256.236 billion, and 52.072 billion yuan, respectively. Among them, the stoma care costs the most in states 1 and 3, urinary catheterization costs the most in state 2, and the drug administration costs the most for state 5. In addition, in the area of daily care services, meal help services are more expensive and have the highest cost, reaching 208.093; in terms of primary care services, the costs of pressure ulcer prevention care and Assist in turning and tapping to expel sputum both growing faster and higher, reaching 222.092 and 210.085 respectively; in terms of professional medical care, Stoma care is the most expensive, reaching 243.641; in the area of ancillary monitoring, blood glucose monitoring costs are higher at 38.229; in terms of spiritual comfort, hospice care was the most expensive, at 87.407; in regards to healthcare, Rehab Physical Therapy cost the most, reaching 221.850; in Senior Education, Security guidance cost the most, at 24.391. (annual cost unit: billion RMB).

**Table 9:** Forecast analysis of the cost of each program for community home-based care services from 2020 to 2035 (billion RMB)

Service content	Items	2020	2025	2030	2035
Daily life care	Bathing assistance	16.318	19.884	24.111	27.666
	Meal help	122.739	149.558	181.348	208.093
	Housekeeping	81.898	100.231	121.438	139.751
Primary Care	Intramuscular injection	60.635	74.208	89.909	103.467
	Dressing change (medicine)	62.007	75.887	91.944	105.809
	Intravenous infusion	38.199	46.750	56.641	65.183
	Blood specimen collection	12.849	15.726	19.053	21.926
	Assist in turning and tapping to expel sputum	123.116	150.675	182.556	210.085
	Pressure ulcer prevention care	130.152	159.287	192.990	222.092
Professional Medical Care Services	Urinary catheterization	146.614	179.434	217.400	250.183
	Bladder Flushing	80.894	98.569	119.521	137.148
	Artificial anal stool bag care	63.547	77.433	93.891	107.738
	Stoma Care	143.707	175.107	212.327	243.641
	Enema	38.715	47.382	57.407	66.064
	Nasal feeding	140.354	171.022	207.373	237.957
	Oxygenation	82.717	101.233	122.652	141.148
Auxiliary monitoring	Blood pressure monitoring	2.224	2.721	3.297	3.794
	Blood glucose Monitoring	22.403	27.419	33.220	38.229
Spiritual comfort	Hospice care	51.556	62.821	76.173	87.407
Healthcare	Rehab Physical Therapy	130.010	159.113	192.779	221.850
	Accompanying to medical appointments	15.048	18.171	22.043	25.196
	Drug administration	75.126	90.716	110.044	125.787
Senior Education	Health consultation	3.938	4.755	5.769	6.594
	Caregiver guidance	1.722	2.079	2.522	2.883
	Legal advice	5.247	6.336	7.685	8.785
	Security guidance	14.567	17.590	21.338	24.391
	Psychological Counseling	2.321	2.802	3.399	3.886
Total	Above all projects	1668.623	2036.910	2468.829	2836.754

#### Total cost analysis by the man-hour costing method

**Fig. 6:**
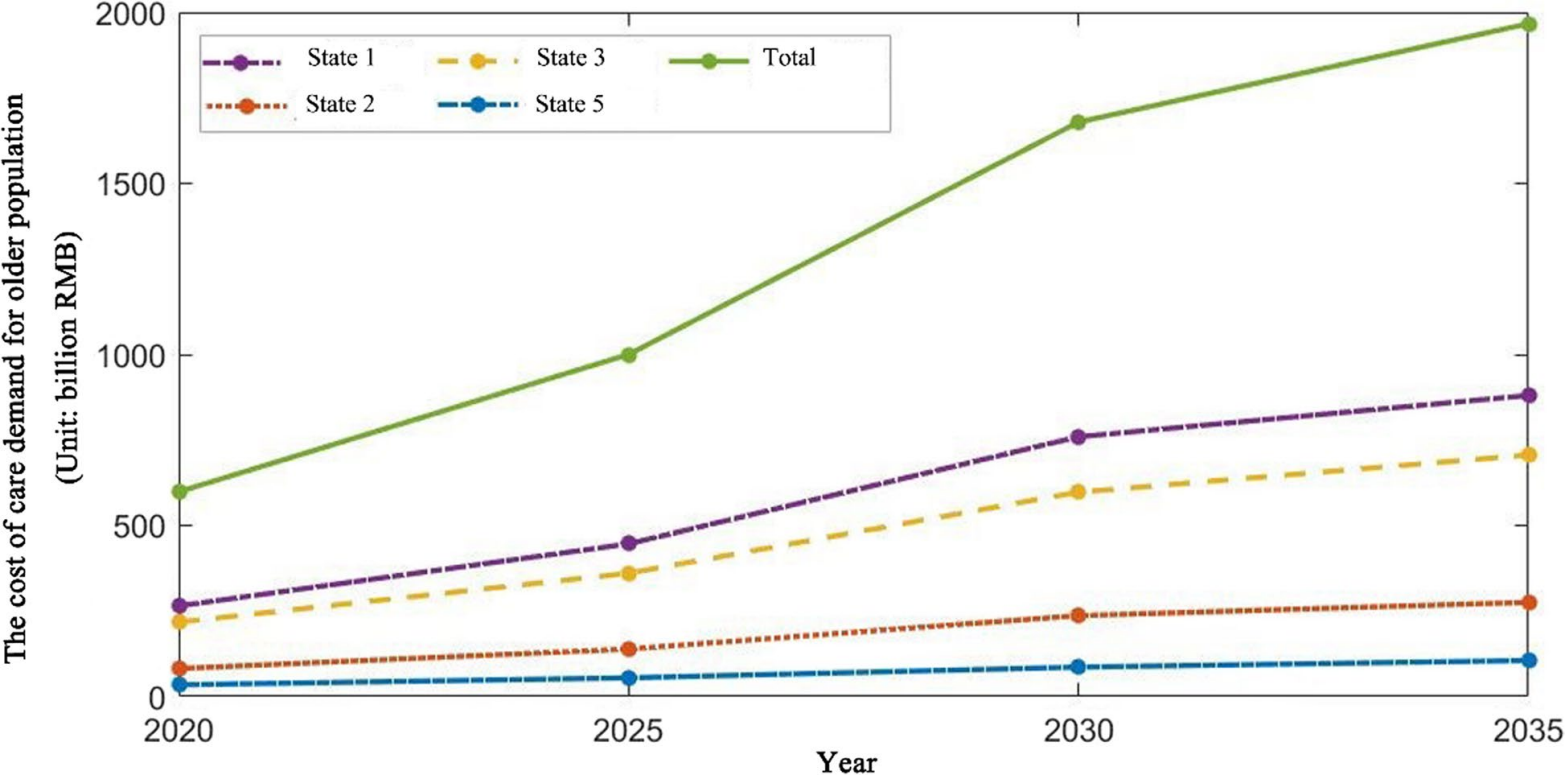
The caregiver needs cost of different functional states for the older population

Tables 10 and 11 show the total costs of care provided by family and professional caregivers, respectively, for different functional states, ages, and genders in community home-based eldercare services from 2020 to 2035. Combined with the man-hour costing method and Markov model prediction analysis, the results show that the total costs of caregivers predicted are increasing from 2020 to 2035. The eldercare costs for those in states 1, 2 ,3, 5 will increase from 264.850 billion, 81.761 billion, 217.484 billion, and 34.227 billion yuan in 2020 to 880.041 billion, 275.369 billion, 705.867 billion, and 105.379 billion yuan in 2035, with growth rates of 207–237%. Especially after 2025, the total costs for the graded care show a rapid upward trend (Fig. 6). Specifically, the cost demands for family caregivers for older adults in care states 1, 2, 3, 5 will increase from 196.418 billion, 60.636 billion, 161.290 billion, and 25.383 billion yuan to 652. 654 billion, 204.219 billion, 523. 483 billion, and 78. 151 billion yuan in 2035, respectively. The cost demands for professional caregivers will increase from 68.432 billion, 21.125 billion, 56.194 billion, and 8.844 billion yuan in 2020 to 227.3387 billion, 71.150 billion, 182.384 billion, and 27.228 billion yuan in 2035, respectively. This indicated that the cost demands of older adults for family caregivers at different functional states are about three times the costs generated by professional caregivers.

Comparing the results of the two cost prediction methods, we found that the project cost method predicts care costs for states 1, 2, 3, 5 to be 1.24, 1.58, 1.78, and 0.49 times higher than the man-hour costing method, respectively. However, the differences in cost predictions between the two methods are similar for different functional states, genders, and ages, except for states 1 and 2, where care costs for individuals aged 60–69 who require other states are higher than for those aged 70 and above. Additionally, the cost of care for males in state 5 is higher than that for females, while the costs for other states are lower for males than for females.

**Table 10:** The total cost of family caregivers’ demand for community home-based older population in different states between 2020 and 2035 (unit: billion RMB)

Age (years)	States	Men				Women			
		2020	2025	2030	2035	2020	2025	2030	2035
**60 ~ 69**	State 1	33.875	52.012	78.881	97.511	65.105	98.745	150.46	185.507
	State 2	10.742	16.381	24.803	30.687	16.375	24.777	37.752	46.536
	State 3	38.4	58.712	88.959	110.041	54.759	82.84	126.246	155.601
	State 5	11.234	16.887	25.536	31.589	7.382	11.077	16.866	20.789
**≥ 70**	State 1	34.102	61.98	90.857	126.782	63.336	118.106	242.854	242.854
	State 2	13.381	24.441	35.851	50.029	20.138	37.443	76.967	76.967
	State 3	28.353	51.697	75.807	105.785	39.778	73.969	152.056	152.056
	State 5	4.058	7.663	11.278	15.745	2.709	4.888	10.028	10.028

**Table 11:** The total cost of professional care demand for community home-based older population in different states between 2020 and 2035 (unit: billion RMB)

Age (years)	States	Men				Women			
		2020	2025	2030	2035	2020	2025	2030	2035
**60 ~ 69**	State 1	11.802	18.121	27.482	33.973	22.683	34.403	52.421	64.631
	State 2	3.742	5.707	8.641	10.692	5.705	8.632	13.153	16.213
	State 3	13.379	20.455	30.994	38.339	19.078	28.862	43.985	54.212
	State 5	3.914	5.883	8.897	11.006	2.572	3.859	5.876	7.243
**≥ 70**	State 1	11.881	21.594	31.655	44.172	22.066	41.149	84.611	84.611
	State 2	4.662	8.515	12.491	17.43	7.016	13.045	26.815	26.815
	State 3	9.878	18.012	26.411	36.856	13.859	25.771	52.977	52.977
	State 5	1.414	2.67	3.929	5.485	0.944	1.703	3.494	3.494

## Discussion

Under the influence of filial piety culture in Confucianism, older adults in China rely on informal care provided by their children and relatives, and the concept of “aging in place” is consistent with the living and living habits of the elderly, so community home-based care is the most suitable way for them to age [51, 52]. This study assessed the demand for community home-based care services for older people with multi-dimensional FD in states 1, 2, 3, and 5, which was higher than in previous research [4]. In fact, older people in pre-disabled (state 5) or disability transition (state 2) states might also have a need for care, such as some cycles of rehabilitation physical therapy services. If the care demands of the people in these categories are not taken into account, the demand and market size of long-term care services for older populations will be underestimated [18]. This study included all possibilities of continuous FD statuses in the care needs and cost assessments, which enhanced the completeness and accuracy of the calculated results. This study assessed and predicted the size and trends of older people in different states of FD. The results showed that the need for community home-based care is growing rapidly. The total size population of older adults with FD states 1, 2, 3, and 5 will rise from 31.915, 52.989, 53.408, 20.985, 82.986 million in 2020 to 54.797, 92.215, 89.569, 33.194, and 132.023 million, respectively, by 2035. Specifically, older adults in states 1, 2, and 3 are the main target groups for community home-based care services, and those in state 5 mainly require healthcare or health consulting services.

Evidence for the potential relationships between age, gender, and transfer probability is scarce. In this study, we found that the probability of transitions in FC statuses varied by gender and the age group of older adults, with male older adults more likely to maintain their statuses or return to better health than females. However, the trend in change is consistent, i.e., the worse the FC statuses, the more difficult it is to return to self-care or better FC statuses. As FC status deteriorates, mortality increases. The possible reason for this is that as age increases, physiological function declines, health problems increase, the ability to recover diminishes, and the probability of different degrees of FD increases, leading to an increased risk of death. This changing trend is also consistent with the findings of other scholars [53–55].

The cost prediction results showed that the market for community home-based care services is large, and will increase at an accelerated rate from 2020 to 2035. Combined with the project cost analysis and Markov model, it is predicted that the eldercare costs will reach 2,836.754 billion yuan by 2035, which is close to the prediction by Zhang et al. [56] that the total demand for home and institutional care for disabled older people in 2035 will be 3,084 billion yuan. However, our study focused on 97% of community home-based older adults with impaired functioning, indicating that the cost of community home-based care is lower than that of a single institutional or home care model. Community home-based care offers the advantage of meeting care needs while also conserving healthcare resources. Our study predicts that the total cost for family and professional caregivers providing community home-based eldercare in China will rise from 598.322 billion to 1,966.656 billion yuan between 2020 and 2035. This is lower than the estimated total cost of care provided by family members and institutional care workers as estimated by Hu et al. [57]. Furthermore, the total cost estimated by both costing methods was significantly lower than the per capita cost of informal care in developed countries such as Sweden and the United States [58, 59]. For example, Chari and colleagues [14] estimated that the total cost of informal care for older adults in the United States is approximately USD 522 billion annually. This highlighted the gap in community home-based care development between China and developed countries. According to a national study in Sweden, approximately 15% of the adult population provides informal care, which is estimated to cost around SEK 152 billion per year. However, replacing informal caregivers with professional care providers would be much more expensive, at around SEK 193.6 billion per year, which is 12.74 times higher than the cost of informal care [59]. Given the significant cost difference between formal and informal care, it is crucial to consider community home-based care as a viable alternative to merely institutional care instead of just complementary care, especially in our national context and economic situation.

A previous study also showed that the provision of more home-based care services was associated with lower demand for nursing visits [60]. Patrick reported that a cost-reducing strategy would only be successful if informal caregiving was a substitute for formal healthcare services. Increased informal caregiving effectively reduces public health care spending by reducing the amount of formal care services [61]. In the future, we should vigorously develop community home-based care services to increase the utilization rate of informal caregivers, such as family caregivers, purchase services that best meet the wishes of the older population at the lowest price, and determine prices that are acceptable to both employers and family members, rather than just protecting eldercare services by increasing the coverage rate and participation rate in long-term care insurance.

In addition, by 2035, the project cost results showed that the cost of urinary catheterization, stoma care, nasal feeding, and pressure ulcer prevention care will be the most among older people with different degrees of FD over time. Among them, the stoma and nasal feeding care would cost the most for patients in states 1 and 3, urinary catheterization will cost the most for people in state 2, and drug administration will cost the most for patients in state 5. Urinary catheterization had the highest cost at 250.183 billion RMB, followed by the cost of stoma care, nasal feeding, and pressure ulcer prevention care. On this basis, we can adjust the proportion of care insurance for different programs according to the cost expenditures of different services projects, and also control the cost appropriately and adjust the price of services. For example, the results showed that the cost of hospice care was relatively high, reaching 87.407 billion RMB in 2035. Many provinces and municipalities in China currently do not have standard pricing for hospice programs, probably because they have overlooked the need for hospice care for the elderly and the importance of this service and are not professionals or systematic in this area. Therefore, more investments should be made in this area in the future.

Furthermore, scholars pointed out that the strategy of replacing institutional care with community home-based care can effectively reduce public healthcare expenditure and care costs, but the improvement of family care services is related to the willingness of care recipients [61]. The government must develop a comprehensive and independent public policy strategy for supporting home-based caregivers and standardizing care cost programs. To achieve this, multi-level and robust needs assessments should be conducted to integrate caregiver support into quality standards for both family care services and professional caregivers, whether publicly funded or private. By drawing on the experience of other countries, a financial compensation system for family caregivers, such as the family care subsidy in Germany and the family care compensation system in the U.S., can be established. A sound caregiving compensation system should cover both the caregiving costs of older adults and the loss of income of family caregivers. To effectively relieve the burden of family caregiving, it is also urgent to raise public awareness of the cost of eldercare. The insurance industry should play a leading role in raising awareness of eldercare as a societal challenge and collaborate with their networks of intermediaries to help the public better understand the potential financial costs and needs associated with becoming a caregiver. It is essential for individuals of all ages to consider how they can financially and psychologically prepare for possible caregiving responsibilities. Additionally, they should become familiar with the support and services available to caregivers. By taking proactive steps to prepare for future caregiving responsibilities and accessing available resources, individuals can more effectively manage the challenges of caregiving while also maintaining their personal well-being and professional obligations.

## Conclusions

This study assessed the demand for the community home-based care of older adults with multi-dimensional FD in states 1, 2, 3, and 5, which was higher compared to previous research. The number of people in these groups will be further increased, and the cost of community home-based care, which includes the cost to family caregivers, will rise accordingly but a vast gap between the cost of family care and professional care will still exist. By 2035, the total cost of services measured by the project cost method was 2836.745 billion, which was 6.086 times less than that measured by the man-hour costing method (17264.498 billion). This indicated that the difference between different measurement methods should be considered when drawing on the forecast results of costs in the future. With regards to each project, the urinary catheterization, stoma care, nasal feeding, and pressure ulcer prevention care cost the most. There exists gender and age differences among the costs for older adults in four FD states. Females had the highest costs in states 1 and 3, at 1.4–1.92 times more than men in each category except for the fifth one. In addition, females aged over 70 in FD states 1, 3, and 5 had lower costs than those aged 60–69, but not those in state 2. The reason for this might be the large reduction in populations aged over 70. And the higher cost for patients in state 2 may be associated with increased hospitalization rates among the oldest patients. However, those aged over 70 in states 1 and 2 had higher community home-based care costs than those aged 60–69. The reason for finding might be the increasing numbers of older male adults in state 1 with poorer home adjustments or care accessibility, even though the male population has declined significantly. Therefore, implementing FD prevention guidelines, and increasing public understanding and awareness of the potential financial costs and needs of becoming a family caregiver are urgent in completing a community home-based care system.

### Strength and limitations

This study offers a novel approach and perspective by projecting the cost of community home-based care for older adults over the next 15 years. We achieved this goal by combining a multi-state Markov model with the man-hour costing method and project cost analysis. Through horizontal and vertical comparisons of service costs for different age and gender groups, we were able to map resource allocation and determine the financial burden of eldercare for older populations in multi-level functional states. Furthermore, by increasing the transparency of costs, particularly the opportunity costs incurred by family caregivers, we can provide valuable insight to the public regarding the potential financial costs and needs associated with becoming a family caregiver. This information can, in turn, serve as a basis for the government to formulate effective eldercare policies.

However, there were still some shortcomings in the study. Firstly, there is a lack of uniform concept regarding the multi-dimensional functional statuses of older adults, particularly in the context of studying multi-level care needs and cost measurements. To address this issue, we adopted Amartya Sen’s “feasible ability” theory and classified the functional capability of older adults into three dimensions: individual, social, and resource-based. Secondly, due to the limited comprehensive implementation of functional capacity assessments for community home-based older adults, the basic data used in this study, including care service cost coefficients, care intensity, and care ratio for older individuals with varying levels of functional capacity, were derived from the standards of selected pilot cities. Consequently, potential biases may exist in the data. To address this issue, we will conduct a large-scale survey to summarize the proportion of care time and cost breakdown required for community home-based care. By combining this data with the classification used in our study, we aim to improve the representativeness of future research and provide more accurate predictions for the cost of care for older adults with multi-dimensional functional conditions.

## Supplementary Information

**Additional file 1: Table S1. **The selection and assigned values in 29 indicators. **Table S2.** Latent class models with fitted metrics and FC probabilities in 2011. **Table S3.** Analysis of LCA of sex-stratified FC in 2011. **Table S4.** Probability transfer matrix of 10-year FC states transfer in older adults by gender and age group (2015-2025). **Table S5.** Probability transfer matrix of 15-year FC states transfer in older adults by gender and age group (2015-2035). **Table S6.** Probability transfer matrix of 20-year FC states transfer in older adults by gender and age group (2015-2035). **Table S7.** Probability matrix of 5-year FC states transfer by gender and age group of sensitive analysis (2015-2020). **Table S8.** Probability matrix of 10-year FC state transfer by gender and age group of sensitive analysis (2015-2025). **Table S9.** Probability matrix of 15-year FC states transfer by gender and age group of sensitive analysis (2015-2030). **Table S10.** Probability matrix of 20-year FC states transfer by gender and age group of sensitive analysis (2015-2035). **Table S11.** The size of older population in different FC states, 2020-2035（million). **Table S12.** Service content for each FD category. **Table S13.** Service frequency coefficient for each service item. **Table S14.** Projected analysis of total program service costs by gender, age group, and FD level, 2020-2035. **Table S15.** Projected Costs for Different FD Levels of Care Services Programs in 2035. **Table S16.** Projected analysis of total program service costs for different levels of FD, 2020-2035.

## Data Availability

CHARLS aims to set up a high quality, nationally representative and publicly available micro-database that provides a wide range of information about the households of the elderly and individual information on the older respondents and their spouses. CHARLS provides broad data that allows for analysis by multiple disciplines. All data stripped of private identifying information will be available for research use at no charge. National baseline data and a baseline national report are public on the CHARLS website and available from http://www.isss.pku.edu.cn/sjsj/charlsxm/index.htm.
