# Supplementary material for: Expenditure projections for community home-based care services for older adults with functional decline in China
Source: Int J Equity Health. 2023 Jul 29;22:143. doi: 10.1186/s12939-023-01954-y (PMC10385915; doi:10.1186/s12939-023-01954-y)
Supplement: Supplementary file 1 — Additional file 1: Table S1. The selection and assigned values in 29 indicators. Table S2. Latent class models with fitted metrics and FC probabilities in 2011. Table S3. Analysis of LCA of sex-stratified FC in 2011. Table S4. Probability transfer matrix of 10-year FC states transfer in older adults by gender and age group (2015-2025). Table S5. Probability transfer matrix of 15-year FC states transfer in older adults by gender and age group (2015-2035). Table S6. Probability transfer matrix of 20-year FC states transfer in older adults by gender and age group (2015-2035). Table S7. Probability matrix of 5-year FC states transfer by gender and age group of sensitive analysis (2015-2020). Table S8. Probability matrix of 10-year FC state transfer by gender and age group of sensitive analysis (2015-2025). Table S9. Probability matrix of 15-year FC states transfer by gender and age group of sensitive analysis (2015-2030). Table S10. Probability matrix of 20-year FC states transfer by gender and age group of sensitive analysis (2015-2035). Table S11. The size of older population in different FC states, 2020-2035（million). Table S12. Service content for each FD category. Table S13. Service frequency coefficient for each service item. Table S14. Projected analysis of total program service costs by gender, age group, and FD level, 2020-2035. Table S15. Projected Costs for Different FD Levels of Care Services Programs in 2035. Table S16. Projected analysis of total program service costs for different levels of FD, 2020-2035. [file 12939_2023_1954_MOESM1_ESM.docx]

**Contents**

[**Table S1.** The selection and assigned values in 29 indicators 1](#_Toc134733198)

[**Table S2.** Latent class models with fitted metrics and FC probabilities in 2011 4](#_Toc134733199)

[**Table S3.** Analysis of LCA of sex-stratified FC in 2011 4](#_Toc134733200)

[**Table S4**. Probability transfer matrix of 10-year FC states transfer in older adults by gender and age group (2015-2025) 5](#_Toc134733201)

[**Table S5**. Probability transfer matrix of 15-year FC states transfer in older adults by gender and age group (2015-2035) 5](#_Toc134733202)

[**Table S6**. Probability transfer matrix of 20-year FC states transfer in older adults by gender and age group (2015-2035) 6](#_Toc134733203)

[**Table S7**. Probability matrix of 5-year FC states transfer by gender and age group of sensitive analysis (2015-2020) 6](#_Toc134733204)

[**Table S8**. Probability matrix of 10-year FC state transfer by gender and age group of sensitive analysis (2015-2025) 7](#_Toc134733205)

[**Table S9**. Probability matrix of 15-year FC states transfer by gender and age group of sensitive analysis (2015-2030) 7](#_Toc134733206)

[**Table S10.** Probability matrix of 20-year FC states transfer by gender and age group of sensitive analysis (2015-2035) 8](#_Toc134733207)

[**Table S11**. The size of older population in different FC states, 2020-2035（million） 9](#_Toc134733208)

[**Table S12.** Service content for each FD category 9](#_Toc134733209)

[**Table S13.** Service frequency coefficient for each service item 10](#_Toc134733210)

[**Table S14**. Projected analysis of total program service costs by gender, age group, and FD level, 2020-2035 11](#_Toc134733211)

[**Table S15.** Projected Costs for Different FD Levels of Care Services Programs in 2035 11](#_Toc134733212)

[**Table S16**. Projected analysis of total program service costs for different levels of FD, 2020-2035 12](#_Toc134733213)

## **Table S1.** The selection and assigned values in 29 indicators

| Dimensions-indicators | Assigned values of 29 indicators |
| --- | --- |
| **Self-care** |  |
| ADL | This refers primarily to the ability to maintain basic life care, including the difficulty of bathing, eating, dressing, using the toilet, controlling urine and feces. The measurement of the difficulty degree of each indicator as follows: “with difficulty but able to complete”, “with difficulty and need help”, “unable to complete” =1; “Without any difficulty” =0. |
| IADL | It is the observation variable of the elderly's social activity ability, which consisted of housework, cooking, shopping and financial management, medicine. The measurement of the difficulty degree of each indicator as follows: “with difficulty but able to complete”, “with difficulty and need help”, “unable to complete” =1; “Without any difficulty” =0. |
| Frailty | Physical Frailty Phenotype was used to measure frailty, which consisted of weight loss, weakness, slowness, low energy expenditure, exhaustion. The score range of PFP scale is 0~5. When the score is 3 and higher, it is defined as frailty=1; otherwise=0. |
| **Motor ability** |  |
| Balance | Stand with the heel side of one foot touching the big toe of the other for 10 seconds: “unable to complete” =1; “Without any difficulty” =0. |
| Falls | Have you fallen in the past two years: falls =1; non-falls =0. |
| Postural transition | Get up after sitting in your chair for a long time:“with difficulty but able to complete”, “with difficulty and need help”, “unable to complete”=1; “Without any difficulty” =0. |
| Arm stretching | Extend your arms up along your shoulders: “with difficulty but able to complete”, “with difficulty and need help”, “unable to complete” =1; “Without any difficulty” =0. |
| Mobility | Climb several flights of stairs without stopping:“with difficulty but able to complete”, “with difficulty and need help”, “unable to complete” =1; “Without any difficulty” =0. |
| **Ability of processing diseases** |  |
| Receiving medical services | In the past month, have you visited a medical institution or received in-patient medical care: have been to=1; not have been to=0. |
| Exceptional treatment | Surgical infusion and acupuncture for any treatment: have been to=1; not have been to=0. |
| Self-treatment | In the past month, have you self-treated yourself in any of the following ways (note: prescriptions are not included here)? “buy over-the-counter or prescription drugs”, “use traditional Chinese herbal medicine”, “take health care products such as vitamins”, “use health care equipment” =0; “none” =1. |
| Assistive Device | Do you use the following AIDS: “crutches”, “walkers”, “manual wheelchairs”, “electric wheelchairs” =1; none of them=0. |
| **Cognitive mental status and communication** |  |
| Cognition | The cognitive function of the elderly is usually measured by three aspects of mental state episodic memory ability and visual spatial ability. CHARLS project team asked interviewees to answer five mathematical calculation questions whether they knew the date of the year or month at the time of interview. One point was scored for each correct answer to the questions of the week and season, and the total score was the mental state score (range: 0-10 points). By asking respondents 10 words and asking them to recall the words at two different times, 0.5 points were scored for each pair of words recalled. The mean value of the two recall scores was the score of episodic memory ability (range :0~10 points). Visual spatial ability measured by pressing the figure painting, painting for 1 point, otherwise don't score (range: 0 ~ 1). Cognitive function in the elderly by three aspects including mental state, Episodic memory ability and visual spatial ability scored (range: 0 ~ 21 points). When the score was equal to or higher than the average (9.7 points), participants had higher cognitive functions, otherwise had low cognitive function. In this paper, below 9.7 points=1; higher than 9.7=0. |
| Memory | How do you feel about your memory now: “excellent”, “very good”, “good”, “general” =0; “Poor” =1. |
| Vision | Including near object vision and distant vision: “Excellent”, “very good”, “good”, “so-so” =0; “bad” =1. |
| Hearing | How do you feel about your hearing: “Excellent”, “very good”, “good”, “so-so” =0; “bad” =1. |
| Sadness | To feel sad, depressed, or depressed: “No”, “mild”, “moderate” =0; “serious”, “very serious” =1. |
| Depression | The Center for Epidemiologic Studies Depression Scale (CES-D), was used in this paper. CES-D consists of 10 items, in which respondents are asked about their feelings and behaviors in the past week, and are given 0~3 points according to the depression degree reflected in the items from low to high, with a total score of 0~30 points. The higher the score is, the more serious the depression is, and generally 10 points or above are considered as having depressive symptoms. a score of 0~9 was defined as no depressive symptoms=0; a score of 10~30 was defined as having depressive symptoms=1. |
| **Medical condition** |  |
| Chronic disease | Have you been diagnosed with [conditions listed below, read one by one] by a doctor: hypertension, Diabetes, Dyslipidemia, Cancer or malignant tumor, Chronic lung diseases, Liver disease, Heart attack, Stroke, Kidney disease, Stomach or other digestive disease, Emotional, nervous, or psychiatric problems, Memory-related disease, Arthritis or rheumatism, Asthma =1; none=0. |
| Physical disabilities | Whether you have a physical disability: yes=1; none=0. |
| **Sources of care** |  |
| Access of caregiver | If you need to be taken care of in daily life, such as eating, dressing, you will have a family member (other than your spouse) or a friend who can take care of you in the long term: no=1; yes=0. |
| Living arrangement | Living arrangement: “live with their children in a same house”, “in a same dwelling or courtyard” =0; “in a same village”, “in another household in this city” “in another province” =1. |
| **Home settings** |  |
| Elevator in a four-story apartment | Housing types: without elevator=1; with elevator=0. |
| Accessibility | Do you have wheelchair accessible around your place: no=1; yes=0. |
| **Social interactions** |  |
| Interacted with friends | Have you done any of this activity in the last month: no=1; yes=0. |
| Community club | Have you done any of this activity in the last month: no=1; yes=0. |
| Provide help to friends | Have you done any of this activity in the last month: no=1; yes=0. |
| Outdoor activities | Have you done any of this activity in the last month: no=1; yes=0. |
| Community-related organization | Have you done any of this activity in the last month: no=1; yes=0. |

## **Table S2.** Latent class models with fitted metrics and FC probabilities in 2011

| **Model** | **Model** | **AIC** | **BIC** | **SSA-BIC** | **Entropy** | **LMR-A *p*-value** | **BLRT *p*-value** | **Classification probability** |
| --- | --- | --- | --- | --- | --- | --- | --- | --- |
| 2011 | 5 | 192196.131 | 193629.541 | 192949.509 | 0.823 | <0.001 | <0.001 | .114/.103/.077/.340/.366 |
| 2013 | 5 | 141486.905 | 142433.500 | 141975.915 | 0.889 | <0.001 | <0.001 | .111/.222/.092/.112/.463 |
| 2015 | 5 | 129808.661 | 130778.856 | 130305.386 | 0.812 | 0.382 | <0.001 | .098/.145/.293/.111/.354 |

*Note*. BIC, Bayesian Information Criterion; SSA-BIC, Sample-Size Adjusted BIC; AIC, Akaike Information Criterion; LMRA-A, Lo-Mendell-Rubin adjusted likelihood ratio test; BLRT, bootstrap likelihood ratio test.

## **Table S3.** Analysis of LCA of sex-stratified FC in 2011

|  | Male | | | | |  |  | Female | | | |
| --- | --- | --- | --- | --- | --- | --- | --- | --- | --- | --- | --- |
|  | State 1 | State 2 | State 3 | State 4 | State 5 |  | State 1 | State 2 | State 3 | Class 4 | Class 5 |
| N (%) | 254(8.5) | 318(10.6) | 174(5.8) | 1257(42) | 991(33.1) |  | 431(14.4) | 300(10.0) | 289(9.6) | 779(26.0) | 1199(40.0) |

*Note.* state 1: viability disorder; state 2: acute disease; state 3: somatic functional disorder; state 4: health; state 5: sub-disorder status.

## **Table S4**. Probability transfer matrix of 10-year FC states transfer in older adults by gender and age group with three times follow-up (2015-2025)

|  |  |  |  |  | Male |  |  |  |  |  |  | Female |  |  |
| --- | --- | --- | --- | --- | --- | --- | --- | --- | --- | --- | --- | --- | --- | --- |
| Age | FC statuses | State1 | State2 | State3 | State4 | State5 | State6 |  | State 1 | State 2 | State 3 | State 4 | State 5 | State 6 |
| 60-69 year | |  |  |  |  |  |  |  |  |  |  |  |  |  |
|  | State 1 | 0.059 | 0.099 | 0.135 | 0.088 | 0.370 | 0.248 |  | 0.131 | 0.176 | 0.223 | 0.081 | 0.289 | 0.101 |
|  | State 2 | 0.059 | 0.099 | 0.136 | 0.090 | 0.377 | 0.239 |  | 0.130 | 0.175 | 0.221 | 0.081 | 0.289 | 0.105 |
|  | State 3 | 0.058 | 0.098 | 0.133 | 0.088 | 0.368 | 0.255 |  | 0.130 | 0.175 | 0.221 | 0.081 | 0.289 | 0.105 |
|  | State 4 | 0.063 | 0.107 | 0.145 | 0.101 | 0.418 | 0.167 |  | 0.131 | 0.177 | 0.224 | 0.085 | 0.302 | 0.082 |
|  | State 5 | 0.062 | 0.106 | 0.144 | 0.099 | 0.412 | 0.177 |  | 0.131 | 0.177 | 0.224 | 0.084 | 0.300 | 0.084 |
|  | State 6 | 0.000 | 0.000 | 0.000 | 0.000 | 0.000 | 1.000 |  | 0.000 | 0.000 | 0.000 | 0.000 | 0.000 | 1.000 |
| ≥70 year | |  |  |  |  |  |  |  |  |  |  |  |  |  |
|  | State 1 | 0.061 | 0.130 | 0.104 | 0.034 | 0.149 | 0.522 |  | 0.118 | 0.201 | 0.151 | 0.026 | 0.097 | 0.408 |
|  | State 2 | 0.063 | 0.133 | 0.107 | 0.036 | 0.154 | 0.509 |  | 0.120 | 0.205 | 0.153 | 0.026 | 0.099 | 0.396 |
|  | State 3 | 0.061 | 0.130 | 0.104 | 0.034 | 0.150 | 0.520 |  | 0.120 | 0.205 | 0.153 | 0.026 | 0.099 | 0.396 |
|  | State 4 | 0.075 | 0.160 | 0.128 | 0.045 | 0.194 | 0.398 |  | 0.129 | 0.220 | 0.165 | 0.029 | 0.110 | 0.347 |
|  | State 5 | 0.074 | 0.157 | 0.126 | 0.043 | 0.189 | 0.411 |  | 0.129 | 0.221 | 0.165 | 0.029 | 0.109 | 0.347 |
|  | State 6 | 0.000 | 0.000 | 0.000 | 0.000 | 0.000 | 1.000 |  | 0.000 | 0.000 | 0.000 | 0.000 | 0.000 | 1.000 |

*Note.* FC: functional capability; state 1: viability disorder; state 2: acute disease; state 3: somatic functional disorder; state 4: health; state 5: sub-disorder status; state 6: death.

## **Table S5**. Probability transfer matrix of 15-year FC states transfer in older adults by gender and age group with three times follow-up (2015-2035)

|  |  |  |  |  | Male |  |  |  |  |  |  | Female |  |  |
| --- | --- | --- | --- | --- | --- | --- | --- | --- | --- | --- | --- | --- | --- | --- |
| Age | FC statuses | State1 | State2 | State3 | State4 | State5 | State6 |  | State 1 | State 2 | State 3 | State 4 | State 5 | State 6 |
| 60-69 year | |  |  |  |  |  |  |  |  |  |  |  |  |  |
|  | State 1 | 0.052 | 0.087 | 0.118 | 0.080 | 0.334 | 0.329 |  | 0.123 | 0.166 | 0.211 | 0.078 | 0.277 | 0.145 |
|  | State 2 | 0.053 | 0.088 | 0.120 | 0.081 | 0.338 | 0.321 |  | 0.123 | 0.166 | 0.210 | 0.077 | 0.276 | 0.148 |
|  | State 3 | 0.051 | 0.086 | 0.117 | 0.079 | 0.331 | 0.336 |  | 0.123 | 0.166 | 0.210 | 0.077 | 0.276 | 0.148 |
|  | State 4 | 0.057 | 0.096 | 0.131 | 0.089 | 0.371 | 0.256 |  | 0.126 | 0.170 | 0.215 | 0.080 | 0.284 | 0.127 |
|  | State 5 | 0.056 | 0.095 | 0.129 | 0.088 | 0.367 | 0.265 |  | 0.125 | 0.169 | 0.215 | 0.079 | 0.283 | 0.128 |
|  | State 6 | 0.000 | 0.000 | 0.000 | 0.000 | 0.000 | 1.000 |  | 0.000 | 0.000 | 0.000 | 0.000 | 0.000 | 1.000 |
| ≥70 year | |  |  |  |  |  |  |  |  |  |  |  |  |  |
|  | State 1 | 0.044 | 0.093 | 0.075 | 0.025 | 0.110 | 0.654 |  | 0.092 | 0.157 | 0.118 | 0.020 | 0.077 | 0.537 |
|  | State 2 | 0.045 | 0.096 | 0.077 | 0.026 | 0.113 | 0.644 |  | 0.094 | 0.160 | 0.120 | 0.021 | 0.078 | 0.528 |
|  | State 3 | 0.044 | 0.094 | 0.075 | 0.025 | 0.110 | 0.652 |  | 0.094 | 0.160 | 0.120 | 0.021 | 0.078 | 0.528 |
|  | State 4 | 0.055 | 0.117 | 0.094 | 0.032 | 0.139 | 0.563 |  | 0.102 | 0.173 | 0.130 | 0.022 | 0.085 | 0.489 |
|  | State 5 | 0.054 | 0.115 | 0.092 | 0.031 | 0.136 | 0.573 |  | 0.102 | 0.173 | 0.130 | 0.022 | 0.085 | 0.489 |
|  | State 6 | 0.000 | 0.000 | 0.000 | 0.000 | 0.000 | 1.000 |  | 0.000 | 0.000 | 0.000 | 0.000 | 0.000 | 1.000 |

*Note.* FC: functional capability; state 1: viability disorder; state 2: acute disease; state 3: somatic functional disorder; state 4: health; state 5: sub-disorder status; state 6: death.

## **Table S6**. Probability transfer matrix of 20-year FC states transfer in older adults by gender and age group with three times follow-up (2015-2035)

|  |  |  |  |  | Male |  |  |  |  |  |  | Female |  |  |
| --- | --- | --- | --- | --- | --- | --- | --- | --- | --- | --- | --- | --- | --- | --- |
| Age | FC statuses | State1 | State2 | State3 | State4 | State5 | State6 |  | State 1 | State 2 | State 3 | State 4 | State 5 | State 6 |
| 60-69 year | |  |  |  |  |  |  |  |  |  |  |  |  |  |
|  | State 1 | 0.046 | 0.078 | 0.106 | 0.072 | 0.299 | 0.401 |  | 0.117 | 0.158 | 0.200 | 0.074 | 0.264 | 0.186 |
|  | State 2 | 0.046 | 0.079 | 0.107 | 0.073 | 0.302 | 0.394 |  | 0.117 | 0.157 | 0.199 | 0.074 | 0.263 | 0.190 |
|  | State 3 | 0.045 | 0.077 | 0.104 | 0.071 | 0.296 | 0.407 |  | 0.117 | 0.157 | 0.199 | 0.074 | 0.263 | 0.190 |
|  | State 4 | 0.051 | 0.086 | 0.117 | 0.080 | 0.331 | 0.335 |  | 0.120 | 0.161 | 0.205 | 0.076 | 0.270 | 0.169 |
|  | State 5 | 0.050 | 0.085 | 0.116 | 0.079 | 0.327 | 0.343 |  | 0.119 | 0.161 | 0.204 | 0.075 | 0.269 | 0.171 |
|  | State 6 | 0.000 | 0.000 | 0.000 | 0.000 | 0.000 | 1.000 |  | 0.000 | 0.000 | 0.000 | 0.000 | 0.000 | 1.000 |
| ≥70 year | |  |  |  |  |  |  |  |  |  |  |  |  |  |
|  | State 1 | 0.032 | 0.068 | 0.054 | 0.018 | 0.080 | 0.749 |  | 0.072 | 0.123 | 0.092 | 0.016 | 0.060 | 0.638 |
|  | State 2 | 0.033 | 0.069 | 0.056 | 0.019 | 0.082 | 0.742 |  | 0.073 | 0.125 | 0.094 | 0.016 | 0.061 | 0.630 |
|  | State 3 | 0.032 | 0.068 | 0.054 | 0.018 | 0.080 | 0.748 |  | 0.073 | 0.125 | 0.094 | 0.016 | 0.061 | 0.630 |
|  | State 4 | 0.040 | 0.085 | 0.068 | 0.023 | 0.100 | 0.683 |  | 0.080 | 0.136 | 0.101 | 0.017 | 0.066 | 0.600 |
|  | State 5 | 0.039 | 0.083 | 0.067 | 0.022 | 0.098 | 0.690 |  | 0.080 | 0.136 | 0.101 | 0.017 | 0.066 | 0.600 |
|  | State 6 | 0.000 | 0.000 | 0.000 | 0.000 | 0.000 | 1.000 |  | 0.000 | 0.000 | 0.000 | 0.000 | 0.000 | 1.000 |

*Note.* FC: functional capability; state 1: viability disorder; state 2: acute disease; state 3: somatic functional disorder; state 4: health; state 5: sub-disorder status; state 6: death.

## **Table S7**. Probability matrix of 5-year FC states transfer by gender and age group of sensitive analysis with two times follow-up (2015-2020)

|  |  |  |  | Male |  |  |  |  |  |  | Female |  |  |
| --- | --- | --- | --- | --- | --- | --- | --- | --- | --- | --- | --- | --- | --- |
| FC statuses | State1 | State2 | State3 | State4 | State5 | State6 |  | State 1 | State 2 | State 3 | State 4 | State 5 | State 6 |
| 60-69 year |  |  |  |  |  |  |  |  |  |  |  |  |  |
| State 1 | 0.084 | 0.131 | 0.179 | 0.086 | 0.375 | 0.146 |  | 0.155 | 0.199 | 0.249 | 0.072 | 0.269 | 0.057 |
| State 2 | 0.076 | 0.123 | 0.167 | 0.093 | 0.396 | 0.145 |  | 0.146 | 0.192 | 0.240 | 0.077 | 0.283 | 0.061 |
| State 3 | 0.078 | 0.124 | 0.169 | 0.089 | 0.385 | 0.156 |  | 0.147 | 0.193 | 0.240 | 0.077 | 0.282 | 0.061 |
| State 4 | 0.059 | 0.109 | 0.142 | 0.123 | 0.496 | 0.071 |  | 0.119 | 0.173 | 0.215 | 0.103 | 0.352 | 0.039 |
| State 5 | 0.062 | 0.111 | 0.147 | 0.119 | 0.481 | 0.080 |  | 0.124 | 0.177 | 0.220 | 0.098 | 0.341 | 0.040 |
| State 6 | 0.000 | 0.000 | 0.000 | 0.000 | 0.000 | 1.000 |  | 0.000 | 0.000 | 0.000 | 0.000 | 0.000 | 1.000 |
| ≥70 year |  |  |  |  |  |  |  |  |  |  |  |  |  |
| State 1 | 0.092 | 0.192 | 0.156 | 0.039 | 0.182 | 0.339 |  | 0.155 | 0.259 | 0.195 | 0.028 | 0.111 | 0.251 |
| State 2 | 0.093 | 0.194 | 0.158 | 0.042 | 0.193 | 0.321 |  | 0.157 | 0.263 | 0.199 | 0.030 | 0.117 | 0.234 |
| State 3 | 0.092 | 0.193 | 0.156 | 0.041 | 0.189 | 0.329 |  | 0.157 | 0.263 | 0.199 | 0.030 | 0.116 | 0.234 |
| State 4 | 0.088 | 0.192 | 0.154 | 0.075 | 0.305 | 0.189 |  | 0.148 | 0.268 | 0.195 | 0.049 | 0.171 | 0.180 |
| State 5 | 0.090 | 0.198 | 0.159 | 0.069 | 0.286 | 0.199 |  | 0.154 | 0.266 | 0.201 | 0.045 | 0.159 | 0.175 |
| State 6 | 0.000 | 0.000 | 0.000 | 0.000 | 0.000 | 1.000 |  | 0.000 | 0.000 | 0.000 | 0.000 | 0.000 | 1.000 |

*Note.* FC: functional capability; state 1: viability disorder; state 2: acute disease; state 3: somatic functional disorder; state 4: health; state 5: sub-disorder status; state 6: death.

## **Table S8**. Probability matrix of 10-year FC state transfer by gender and age group of sensitive analysis with two times follow-up (2015-2025)

|  |  |  |  | Male |  |  |  |  |  |  | Female |  |  |
| --- | --- | --- | --- | --- | --- | --- | --- | --- | --- | --- | --- | --- | --- |
| FC statuses | State1 | State2 | State3 | State4 | State5 | State6 |  | State 1 | State 2 | State 3 | State 4 | State 5 | State 6 |
| 60-69 year |  |  |  |  |  |  |  |  |  |  |  |  |  |
| State 1 | 0.059 | 0.099 | 0.135 | 0.088 | 0.370 | 0.248 |  | 0.131 | 0.176 | 0.223 | 0.081 | 0.289 | 0.101 |
| State 2 | 0.059 | 0.099 | 0.136 | 0.090 | 0.376 | 0.239 |  | 0.130 | 0.175 | 0.221 | 0.081 | 0.289 | 0.105 |
| State 3 | 0.058 | 0.098 | 0.133 | 0.088 | 0.368 | 0.255 |  | 0.130 | 0.175 | 0.221 | 0.081 | 0.289 | 0.105 |
| State 4 | 0.063 | 0.107 | 0.145 | 0.101 | 0.418 | 0.167 |  | 0.130 | 0.177 | 0.224 | 0.085 | 0.301 | 0.082 |
| State 5 | 0.062 | 0.106 | 0.144 | 0.099 | 0.412 | 0.176 |  | 0.131 | 0.177 | 0.224 | 0.084 | 0.300 | 0.084 |
| State 6 | 0.000 | 0.000 | 0.000 | 0.000 | 0.000 | 1.000 |  | 0.000 | 0.000 | 0.000 | 0.000 | 0.000 | 1.000 |
| ≥70 year |  |  |  |  |  |  |  |  |  |  |  |  |  |
| State 1 | 0.061 | 0.130 | 0.104 | 0.034 | 0.149 | 0.522 |  | 0.118 | 0.201 | 0.150 | 0.026 | 0.097 | 0.408 |
| State 2 | 0.063 | 0.133 | 0.107 | 0.035 | 0.154 | 0.509 |  | 0.120 | 0.205 | 0.153 | 0.026 | 0.099 | 0.396 |
| State 3 | 0.061 | 0.130 | 0.104 | 0.034 | 0.150 | 0.520 |  | 0.120 | 0.205 | 0.153 | 0.026 | 0.099 | 0.396 |
| State 4 | 0.075 | 0.160 | 0.128 | 0.045 | 0.194 | 0.398 |  | 0.129 | 0.220 | 0.165 | 0.029 | 0.110 | 0.347 |
| State 5 | 0.074 | 0.157 | 0.126 | 0.043 | 0.189 | 0.411 |  | 0.129 | 0.221 | 0.165 | 0.029 | 0.109 | 0.347 |
| State 6 | 0.000 | 0.000 | 0.000 | 0.000 | 0.000 | 1.000 |  | 0.000 | 0.000 | 0.000 | 0.000 | 0.000 | 1.000 |

*Note.* FC: functional capability; state 1: viability disorder; state 2: acute disease; state 3: somatic functional disorder; state 4: health; state 5: sub-disorder status; state 6: death.

## **Table S9**. Probability matrix of 15-year FC states transfer by gender and age group of sensitive analysis with two times follow-up (2015-2030)

|  |  |  |  | Male |  |  |  |  |  |  | Female |  |  |
| --- | --- | --- | --- | --- | --- | --- | --- | --- | --- | --- | --- | --- | --- |
| FC statuses | State1 | State2 | State3 | State4 | State5 | State6 |  | State 1 | State 2 | State 3 | State 4 | State 5 | State 6 |
| 60-69 year |  |  |  |  |  |  |  |  |  |  |  |  |  |
| State 1 | 0.052 | 0.087 | 0.118 | 0.080 | 0.334 | 0.329 |  | 0.123 | 0.166 | 0.211 | 0.078 | 0.277 | 0.145 |
| State 2 | 0.052 | 0.088 | 0.120 | 0.081 | 0.338 | 0.321 |  | 0.123 | 0.166 | 0.210 | 0.077 | 0.276 | 0.148 |
| State 3 | 0.051 | 0.086 | 0.117 | 0.079 | 0.331 | 0.336 |  | 0.123 | 0.165 | 0.210 | 0.077 | 0.276 | 0.148 |
| State 4 | 0.057 | 0.096 | 0.131 | 0.089 | 0.371 | 0.256 |  | 0.126 | 0.170 | 0.215 | 0.080 | 0.284 | 0.127 |
| State 5 | 0.056 | 0.095 | 0.129 | 0.088 | 0.367 | 0.264 |  | 0.125 | 0.169 | 0.214 | 0.079 | 0.283 | 0.128 |
| State 6 | 0.000 | 0.000 | 0.000 | 0.000 | 0.000 | 1.000 |  | 0.000 | 0.000 | 0.000 | 0.000 | 0.000 | 1.000 |
| ≥70 year |  |  |  |  |  |  |  |  |  |  |  |  |  |
| State 1 | 0.044 | 0.093 | 0.075 | 0.025 | 0.110 | 0.654 |  | 0.092 | 0.157 | 0.118 | 0.020 | 0.077 | 0.537 |
| State 2 | 0.045 | 0.096 | 0.077 | 0.026 | 0.113 | 0.644 |  | 0.094 | 0.160 | 0.120 | 0.021 | 0.078 | 0.527 |
| State 3 | 0.044 | 0.094 | 0.075 | 0.025 | 0.110 | 0.652 |  | 0.094 | 0.160 | 0.120 | 0.021 | 0.078 | 0.528 |
| State 4 | 0.055 | 0.117 | 0.094 | 0.032 | 0.139 | 0.563 |  | 0.102 | 0.173 | 0.130 | 0.022 | 0.085 | 0.489 |
| State 5 | 0.054 | 0.115 | 0.092 | 0.031 | 0.136 | 0.573 |  | 0.102 | 0.173 | 0.130 | 0.022 | 0.085 | 0.489 |
| State 6 | 0.000 | 0.000 | 0.000 | 0.000 | 0.000 | 1.000 |  | 0.000 | 0.000 | 0.000 | 0.000 | 0.000 | 1.000 |

*Note.* FC: functional capability; state 1: viability disorder; state 2: acute disease; state 3: somatic functional disorder; state 4: health; state 5: sub-disorder status; state 6: death.

## **Table S10.** Probability matrix of 20-year FC states transfer by gender and age group of sensitive analysis with two times follow-up (2015-2035)

|  |  |  |  | Male |  |  |  |  |  |  | Female |  |  |
| --- | --- | --- | --- | --- | --- | --- | --- | --- | --- | --- | --- | --- | --- |
| FC statuses | State1 | State2 | State3 | State4 | State5 | State6 |  | State 1 | State 2 | State 3 | State 4 | State 5 | State 6 |
| 60-69 year |  |  |  |  |  |  |  |  |  |  |  |  |  |
| State 1 | 0.046 | 0.078 | 0.105 | 0.072 | 0.299 | 0.401 |  | 0.117 | 0.158 | 0.200 | 0.074 | 0.264 | 0.186 |
| State 2 | 0.046 | 0.079 | 0.107 | 0.073 | 0.302 | 0.394 |  | 0.117 | 0.157 | 0.199 | 0.074 | 0.263 | 0.190 |
| State 3 | 0.045 | 0.077 | 0.104 | 0.071 | 0.296 | 0.407 |  | 0.117 | 0.157 | 0.199 | 0.074 | 0.263 | 0.190 |
| State 4 | 0.051 | 0.086 | 0.117 | 0.080 | 0.331 | 0.335 |  | 0.120 | 0.161 | 0.204 | 0.076 | 0.270 | 0.170 |
| State 5 | 0.050 | 0.085 | 0.116 | 0.079 | 0.327 | 0.343 |  | 0.119 | 0.161 | 0.204 | 0.075 | 0.269 | 0.171 |
| State 6 | 0.000 | 0.000 | 0.000 | 0.000 | 0.000 | 1.000 |  | 0.000 | 0.000 | 0.000 | 0.000 | 0.000 | 1.000 |
| ≥70 year |  |  |  |  |  |  |  |  |  |  |  |  |  |
| State 1 | 0.032 | 0.068 | 0.054 | 0.018 | 0.080 | 0.749 |  | 0.072 | 0.123 | 0.092 | 0.016 | 0.060 | 0.637 |
| State 2 | 0.033 | 0.069 | 0.056 | 0.019 | 0.082 | 0.742 |  | 0.073 | 0.125 | 0.093 | 0.016 | 0.061 | 0.630 |
| State 3 | 0.032 | 0.068 | 0.054 | 0.018 | 0.080 | 0.748 |  | 0.073 | 0.125 | 0.094 | 0.016 | 0.061 | 0.630 |
| State 4 | 0.040 | 0.085 | 0.068 | 0.023 | 0.100 | 0.683 |  | 0.079 | 0.136 | 0.101 | 0.017 | 0.066 | 0.600 |
| State 5 | 0.039 | 0.083 | 0.067 | 0.022 | 0.098 | 0.690 |  | 0.080 | 0.136 | 0.101 | 0.017 | 0.066 | 0.600 |
| State 6 | 0.000 | 0.000 | 0.000 | 0.000 | 0.000 | 1.000 |  | 0.000 | 0.000 | 0.000 | 0.000 | 0.000 | 1.000 |

*Note.* FC: functional capability; state 1: viability disorder; state 2: acute disease; state 3: somatic functional disorder; state 4: health; state 5: sub-disorder status; state 6: death.

## **Table S11**. The size of older population in different FC states, 2020-2035（million）

| FC statuses | 2020 | 2025 | 2030 | 2035 |
| --- | --- | --- | --- | --- |
| State 1 | 31.915 | 39.237 | 47.534 | 54.797 |
| State 2 | 52.989 | 65.723 | 79.436 | 92.215 |
| State 3 | 53.408 | 64.583 | 78.371 | 89.569 |
| State 4 | 20.985 | 24.356 | 29.634 | 33.194 |
| State 5 | 82.986 | 96.678 | 117.668 | 132.023 |

*Note.* FC: functional capability; state 1: viability disorder; state 2: acute disease; state 3: somatic functional disorder; state 4: health; state 5: sub-disorder status.

## **Table S12.** Service content for each FD category

| Service content | Items | State 1 | State 2 | State 3 | State 5 |
| --- | --- | --- | --- | --- | --- |
| Daily life care | Bathing assistance | √ | ○ | √ | ○ |
|  | Meal help | √ | ○ | √ | ○ |
|  | Housekeeping | √ | √ | √ | ○ |
| Primary Care | Intramuscular injection | √ | √ | √ | ○ |
|  | Dressing change (medicine) | √ | √ | √ | ○ |
|  | Intravenous infusion | √ | √ | √ | ○ |
|  | Blood specimen collection | √ | √ | √ | ○ |
|  | Assist in turning and tapping to expel sputum | √ | √ | √ | ○ |
|  | Pressure ulcer prevention care | √ | √ | √ | ○ |
| Professional Medical Care Services | Urinary catheterization | √ | √ | √ | ○ |
|  | Bladder Flushing | √ | ○ | √ | ○ |
|  | Artificial anal stool bag care | √ | ○ | √ | ○ |
|  | Stoma Care | √ | ○ | √ | ○ |
|  | Enema | √ | √ | √ | ○ |
|  | Nasal feeding | √ | ○ | √ | ○ |
|  | Oxygenation | √ | √ | √ | ○ |
| Auxiliary monitoring | Blood pressure monitoring | √ | √ | √ | ○ |
|  | Blood glucose Monitoring | √ | √ | √ | ○ |
| Spiritual comfort | Hospice care | √ | ○ | √ | ○ |
| Healthcare | Rehab Physical Therapy | √ | √ | √ | ○ |
|  | Accompanying to medical appointments | √ | √ | √ | √ |
|  | Drug administration | √ | √ | √ | √ |
| Senior Education | Health consultation | √ | √ | √ | √ |
|  | Caregiver guidance | √ | √ | √ | √ |
|  | Legal advice | √ | √ | √ | √ |
|  | Security guidance | √ | √ | √ | √ |
|  | Psychological Counseling | √ | √ | √ | √ |

## **Table S13.** Service frequency coefficient for each service item

| Service content | Items | State 1 | State 2 | State 3 | State 5 |
| --- | --- | --- | --- | --- | --- |
| Daily life care | Bathing assistance | 0.021 | 0.000 | 0.015 | 0.000 |
|  | Meal help | 0.179 | 0.000 | 0.126 | 0.000 |
|  | Housekeeping | 0.180 | 0.077 | 0.126 | 0.000 |
| Primary Care | Intramuscular injection | 0.108 | 0.046 | 0.076 | 0.000 |
|  | Dressing change (medicine) | 0.108 | 0.046 | 0.076 | 0.000 |
|  | Intravenous infusion | 0.066 | 0.028 | 0.046 | 0.000 |
|  | Blood specimen collection | 0.020 | 0.008 | 0.014 | 0.000 |
|  | Assist in turning and tapping to expel sputum | 0.108 | 0.046 | 0.076 | 0.000 |
|  | Pressure ulcer prevention care | 0.108 | 0.046 | 0.076 | 0.000 |
| Professional Medical Care Services | Urinary catheterization | 0.066 | 0.028 | 0.046 | 0.000 |
|  | Bladder Flushing | 0.108 | 0.000 | 0.076 | 0.000 |
|  | Artificial anal stool bag care | 0.108 | 0.000 | 0.076 | 0.000 |
|  | Stoma Care | 0.108 | 0.000 | 0.076 | 0.000 |
|  | Enema | 0.066 | 0.028 | 0.046 | 0.000 |
|  | Nasal feeding | 0.108 | 0.000 | 0.076 | 0.000 |
|  | Oxygenation | 0.066 | 0.028 | 0.046 | 0.000 |
| Auxiliary monitoring | Blood pressure monitoring | 0.020 | 0.008 | 0.014 | 0.000 |
|  | Blood glucose Monitoring | 0.039 | 0.017 | 0.027 | 0.000 |
| Spiritual comfort | Hospice care | 0.066 | 0.000 | 0.046 | 0.000 |
| Healthcare | Rehab Physical Therapy | 0.138 | 0.059 | 0.097 | 0.000 |
|  | Accompanying to medical appointments | 0.025 | 0.011 | 0.018 | 0.011 |
|  | Drug administration | 0.064 | 0.027 | 0.045 | 0.027 |
| Senior Education | Health consultation | 0.039 | 0.017 | 0.027 | 0.017 |
|  | Caregiver guidance | 0.020 | 0.008 | 0.014 | 0.008 |
|  | Legal advice | 0.020 | 0.008 | 0.014 | 0.008 |
|  | Security guidance | 0.066 | 0.028 | 0.046 | 0.028 |
|  | Psychological Counseling | 0.020 | 0.008 | 0.014 | 0.008 |

*Note.* state 1: viability disorder; state 2: acute disease; state 3: somatic functional disorder; state 5: sub-disorder status.

## **Table S14**. Projected analysis of total program service costs by gender, age group, and FD level, 2020-2035

|  |  | Male |  |  |  |  | Female |  |  |
| --- | --- | --- | --- | --- | --- | --- | --- | --- | --- |
| FD statuses | 2020 | 2025 | 2030 | 2035 |  | 2020 | 2025 | 2030 | 2035 |
| 60-69 |  |  |  |  |  |  |  |  |  |
| State 1 | 109.950 | 123.216 | 151.314 | 163.539 |  | 211.312 | 233.926 | 288.622 | 311.121 |
| State 2 | 44.163 | 49.156 | 60.268 | 65.194 |  | 67.327 | 74.352 | 91.732 | 98.863 |
| State 3 | 178.340 | 199.017 | 244.171 | 264.073 |  | 254.313 | 280.805 | 346.516 | 373.406 |
| State 5 | 14.486 | 15.893 | 19.460 | 21.048 |  | 9.518 | 10.425 | 12.853 | 13.852 |
| ≥70 |  |  |  |  |  |  |  |  |  |
| State 1 | 110.687 | 146.831 | 174.288 | 212.632 |  | 205.571 | 279.794 | 335.293 | 407.300 |
| State 2 | 55.017 | 73.345 | 87.114 | 106.285 |  | 82.797 | 112.362 | 134.618 | 163.513 |
| State 3 | 131.680 | 175.240 | 208.073 | 253.858 |  | 184.737 | 250.736 | 300.410 | 364.899 |
| State 5 | 5.233 | 7.212 | 8.595 | 10.491 |  | 3.493 | 4.601 | 5.501 | 6.681 |

*Note.* FD: functional decline; state 1: viability disorder; state 2: acute disease; state 3: somatic functional disorder; state 5: sub-disorder status.

## **Table S15.** Projected Costs for Different FD Levels of Care Services Programs in 2035

| Service content | Items | Class 1 | Class 2 | Class 3 | Class 5 |
| --- | --- | --- | --- | --- | --- |
| Daily life care | Bathing assistance | 12.882 | 0.000 | 14.784 | 0.000 |
|  | Meal help | 96.892 | 0.000 | 111.201 | 0.000 |
|  | Housekeeping | 48.799 | 34.945 | 56.006 | 0.000 |
| Primary Care | Intramuscular injection | 36.130 | 25.873 | 41.465 | 0.000 |
|  | Dressing change (medicine) | 36.947 | 26.458 | 42.404 | 0.000 |
|  | Intravenous infusion | 22.761 | 16.299 | 26.122 | 0.000 |
|  | Blood specimen collection | 7.656 | 5.483 | 8.787 | 0.000 |
|  | Assist in turning and tapping to expel sputum | 73.359 | 52.533 | 84.193 | 0.000 |
|  | Pressure ulcer prevention care | 77.552 | 55.535 | 89.005 | 0.000 |
| Professional Medical Care Services | Urinary catheterization | 87.361 | 62.560 | 100.262 | 0.000 |
|  | Bladder Flushing | 63.859 | 0.000 | 73.289 | 0.000 |
|  | Artificial anal stool bag care | 50.165 | 0.000 | 57.573 | 0.000 |
|  | Stoma Care | 113.444 | 0.000 | 130.197 | 0.000 |
|  | Enema | 23.069 | 16.520 | 26.476 | 0.000 |
|  | Nasal feeding | 110.797 | 0.000 | 127.159 | 0.000 |
|  | Oxygenation | 49.287 | 35.295 | 56.566 | 0.000 |
| Auxiliary monitoring | Blood pressure monitoring | 1.325 | 0.949 | 1.521 | 0.000 |
|  | Blood glucose Monitoring | 13.349 | 9.559 | 15.321 | 0.000 |
| Spiritual comfort | Hospice care | 40.699 | 0.000 | 46.709 | 0.000 |
| Healthcare | Rehab Physical Therapy | 77.467 | 55.475 | 88.908 | 0.000 |
|  | Accompanying to medical appointments | 6.479 | 4.640 | 7.436 | 6.642 |
|  | Drug administration | 32.344 | 23.162 | 37.121 | 33.160 |
| Senior Education | Health consultation | 1.695 | 1.214 | 1.946 | 1.738 |
|  | Caregiver guidance | 0.741 | 0.531 | 0.851 | 0.760 |
|  | Legal advice | 2.259 | 1.618 | 2.593 | 2.316 |
|  | Security guidance | 6.272 | 4.491 | 7.198 | 6.430 |
|  | Psychological Counseling | 0.999 | 0.715 | 1.147 | 1.024 |
|  | Total | 1094.591 | 433.855 | 1256.236 | 52.071 |

*Note.* state 1: viability disorder; state 2: acute disease; state 3: somatic functional disorder; state 5: sub-disorder status.

## **Table S16**. Projected analysis of total program service costs for different levels of FD, 2020-2035

| FD statuses | 2020 | 2025 | 2030 | 2035 |
| --- | --- | --- | --- | --- |
| State 1 | 637.520 | 783.767 | 949.516 | 1094.591 |
| State 2 | 249.304 | 309.215 | 373.732 | 433.855 |
| State 3 | 749.068 | 905.798 | 1099.172 | 1256.236 |
| State 5 | 32.731 | 38.131 | 46.410 | 52.071 |
| Total | 1668.623 | 2036.910 | 2468.829 | 2836.754 |

*Note.* FD: functional decline; state 1: viability disorder; state 2: acute disease; state 3: somatic functional disorder; state 5: sub-disorder status.
